# Identifying Causal Effects Under Functional Dependencies †

**DOI:** 10.3390/e26121061

**Published:** 2024-12-06

**Authors:** Yizuo Chen, Adnan Darwiche

**Affiliations:** Computer Science Department, University of California, Los Angeles, CA 90095, USA; darwiche@cs.ucla.edu

**Keywords:** causal effects, identifiability, functional dependencies

## Abstract

We study the identification of causal effects, motivated by two improvements to identifiability that can be attained if one knows that some variables in a causal graph are functionally determined by their parents (without needing to know the specific functions). First, an unidentifiable causal effect may become identifiable when certain variables are functional. Secondly, certain functional variables can be excluded from being observed without affecting the identifiability of a causal effect, which may significantly reduce the number of needed variables in observational data. Our results are largely based on an elimination procedure that removes functional variables from a causal graph while preserving key properties in the resulting causal graph, including the identifiability of causal effects. Our treatment of functional dependencies in this context mandates a formal, systematic, and general treatment of positivity assumptions, which are prevalent in the literature on causal effect identifiability and which interact with functional dependencies, leading to another contribution of the presented work.

## 1. Introduction

A causal effect measures the impact of an intervention on some events of interest and is exemplified by the question, “What is the probability that a patient would recover had they taken a drug?”. This type of question, also known as an interventional query, belongs to the second rung of Pearl’s causal hierarchy [1], so it ultimately requires experimental studies if it is to be estimated from data. However, it is well known that such interventional queries can sometimes be answered based on observational queries (first rung of the causal hierarchy), which can be estimated from observational data. This becomes very significant when experimental studies are either not available, expensive to conduct, or would entail ethical concerns. Hence, a key question in causal inference asks when and how a causal effect can be estimated from available observational data, assuming a causal graph is provided [2].

More precisely, given a set of *treatment* variables (X) and a set of *outcome* variables (Y), the causal effect of x on Y, denoted as Pr(Y|do(x)) or ${\mathrm{Pr}}_{x}\left(Y\right)$, is the marginal probability on Y when an intervention sets the states of variables (X) to x. The problem of identifying a causal effect studies whether ${\mathrm{Pr}}_{x}\left(Y\right)$ can be *uniquely* determined from a causal graph and a distribution (Pr(V)) over some variables (V) in the causal graph [2], where Pr(V) is typically estimated from observational data. The casual effect is guaranteed to be identifiable if V corresponds to all variables in the causal graph (with some positivity assumptions), that is, if all variables in the causal graph are observed. When some variables are hidden (unobserved), it is possible that different parameterizations of the causal graph will induce the same distribution (Pr(V)) but different values for the causal effect (${\mathrm{Pr}}_{x}\left(Y\right)$), which leads to unidentifiablility. In the past few decades, a significant amount of effort has been devoted to studying the identifiability of causal effects (see, e.g., [2,3,4,5,6,7]). Some early works include the *back-door criterion* [2,8] and the *front-door criterion* [2,3]. These criteria are sound but incomplete, as they may fail to identify certain causal effects that are, indeed, identifiable. Complete identification methods include the do-calculus method [2], the identification algorithm presented in [9], and the ID algorithm proposed in [10]. These methods require some positivity assumptions (constraints) on the observational distribution (Pr(V)) and can derive an identifying formula that computes the causal effect based on Pr(V) when the causal effect is identifiable. Some recent works take a different approach by first estimating the parameters of a causal graph to obtain a fully specified causal model, which is then used to estimate causal effects through inference [11,12,13,14]. Further works focus on the efficiency of estimating causal effects from finite data, e.g., [15,16,17,18].

One main challenge of these algorithms is that they try to identify causal effects from limited information in the form of a causal graph and data on observed variables (V). This becomes a problem when only a small number of variables (V) is observed, since Pr(V) alone may not provide enough information for deciding the values of causal effects. Such scenarios happen when the collection of data on some variables is infeasible, e.g., if these variables (such as gender and age) involve confidential information. A recent line of work mitigates this problem by studying the impact of additional information on identifiability beyond causal graphs and observational data. For example, Tikka et al. [19] showed that certain unidentifiable causal effects can become identifiable given information about context-specific independence. Our work in this paper follows the same direction, as we consider the problem of causal effect identification in the presence of a particular type of qualitative knowledge called *functional dependencies* [20]. We say there is a functional dependency between a variable (*X*) and its parents (P) in the causal graph if the distribution (Pr(X|P)) is deterministic but we do not know the distribution itself (i.e., the specific values of Pr(x|p)). In this case, we also say that variable *X* is *functional.* Previous works have shown that functional dependencies can be exploited to improve the efficiency of Bayesian network inference [13,21,22,23,24]. We complement these works by showing that functional dependencies can also be exploited to improve the identifiability of causal effects, especially in the presence of hidden variables. In particular, we show that some unidentifiable causal effects may become identifiable, given such functional dependencies; propose techniques for testing identifiability in this context; and highlight other implications of such dependencies on the practice of identifiability.

Consider the following motivational example where we are interested in how the enforcement of speed limits may affect car accidents. The driving age (*A*) is functionally determined by country (*C*), driving age and country are causes of speed (*X*), and speed and driving age are causes of accidents (*Y*). The DAG on the right captures the causal relations among these variables, where variable *A* is circled to indicate it is functional. Furthermore, suppose that variables C,X,Y are observed. According to classical cause–effect identification methods (e.g., do-calculus and ID algorithm), the causal effect of *X* on *Y* is unidentifiable in this case. However, if we take into account that variable *A* is a function of *C*, which restricts the class of distributions under consideration, then the causal effect of *X* on *Y* becomes identifiable. This exemplifies the improvements to identifiability pursued in this paper.

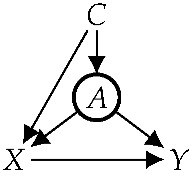



Consider a causal graph (*G*) and a distribution (Pr(V)) over the observed variables (V) in *G*. To check the identifiability of a causal effect, it is standard to first apply the *projection* operation proposed in [25,26], which constructs another causal graph ($G^{\prime }$) with V as its non-root variables, followed by the application of an identification algorithm to $G^{\prime }$, like the ID algorithm [10]. We call this two-stage procedure *project-ID*. One restriction of project-ID is that it is applicable only under some positivity constraints (assumptions), such as strict positivity (Pr(V)>0), which preclude some events from having a zero probability. Nevertheless, these positivity constraints are not always satisfiable in practice and may contradict functional dependencies. For example, if *Y* is a function of *X*, then the positivity constraint (Pr(X,Y)>0) never holds. To systematically treat this interaction between positivity constraints and functional dependencies, we formulate the notion of *constrained identifiability*, which takes positivity constraints as an input (in addition to the causal graph (*G*) and distribution (Pr(V))). We also formulate the notion of *functional identifiability*, which further takes functional dependencies as an input. This allows us to explicitly treat the interactions between positivity constraints and functional dependencies, which is needed for the combination of classical methods like project-ID with the results we present in this paper.

The paper is structured as follows. We start with some technical preliminaries in Section 2. We formally define positivity constraints and functional dependencies in Section 3, where we also introduce the problems of constrained and functional identifiability. Section 4 introduces two primitive operations, *functional elimination* and *functional projection*, which are needed for later treatments. Section 5 presents our core results on functional identifiability and how they can be combined with existing identifiability algorithms. We conduct experiments to evaluate the effectiveness of functional dependencies on cause–effect identifiability in Section 6. Finally, we close with concluding remarks in Section 7. Proofs of all results are included in Appendix C. This paper is an extended version of [27].

## 2. Technical Preliminaries

We consider discrete variables in this work. Single variables are denoted by uppercase letters (e.g., *X*), and their states are denoted by lowercase letters (e.g., *x*). Sets of variables are denoted by bold uppercase letters (e.g., X), and their instantiations (sets of values) are denoted by bold lowercase letters (e.g., x).

### 2.1. Causal Bayesian Networks and Interventions

A Causal Bayesian Network (CBN) is a pair 〈G,F〉, where *G* is a *causal graph* in the form of a directed acyclic graph (DAG) and F is a set of conditional probability tables (CPTs). We have one CPT for each variable (*X*) with parents P in *G*, denoted as $f_{X}\left(X,P\right)$, which specifies the conditional probability distributions (Pr(X|P)). It follows that every CPT ($f_{X}\left(X,P\right)$) satisfies the following properties: $f_{X}\left(x,p\right)\in \left[0,1\right]$ for all instantiations (x,p) and $\sum_{x}f_{X}\left(x,p\right)=1$ for each instantiation (p). For simplicity, we also denote $f_{X}\left(X,P\right)$ as $f_{X}$.

**Figure 1:**
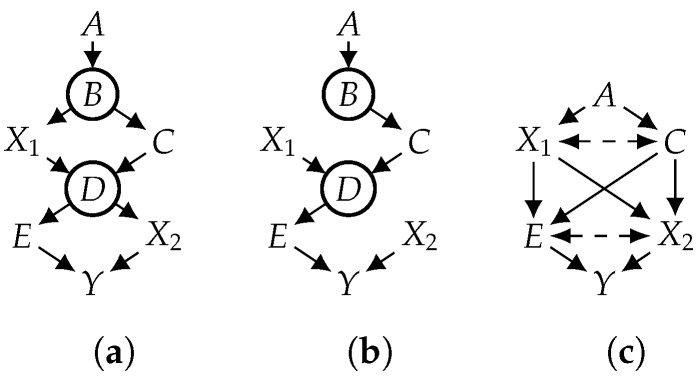
Mutilated and projected graphs of a causal graph. Hidden variables are circled. A bidirected edge ($V_{1}⤎⤏V_{2}$) is aa compact notation for $V_{1}\leftarrow H\to V_{2}$, where *H* is an auxiliary hidden variable. (**a**) Causal graph; (**b**) mutilated graph; (**c**) projected graph.

A CBN induces a joint distribution over its variables (V), which is exactly the product of its CPTs, i.e., $\mathrm{Pr}\left(V\right)=\prod_{V\in V}f_{V}$. In the CBN shown in Figure 1a, for example, $\mathrm{Pr}\left(A,B,X_{1},C,D,E,X_{2},Y\right)=f_{A}\left(A\right)$$f_{B}\left(B,A\right)$$f_{X_{1}}\left(X_{1},B\right)$$f_{C}\left(B,C\right)$$f_{D}\left(D,X_{1},C\right)$$f_{E}\left(E,D\right)$$f_{X_{2}}\left(X_{2},D\right)$$f_{Y}\left(Y,E,X_{2}\right)$. Applying a treatment (do(x)) to the joint distribution yields a new distribution called the *interventional distribution*, denoted as ${\mathrm{Pr}}_{x}\left(V\right)$. One way to compute the interventional distribution is to consider the *mutilated CBN* $\langle G^{\prime },F^{\prime }\rangle$ that is constructed from the original CBN 〈G,F〉 as follows: Remove from *G* all edges that point to variables in X; then, replace the CPT in F for each X∈X with a CPT ($f_{X}\left(X\right)$ where $f_{X}\left(x\right)=1$ if *x* is consistent with x and $f_{X}\left(x\right)=0$ otherwise). Figure 1a depicts a causal graph (*G*), and Figure 1b depicts the mutilated causal graph ($G^{\prime }$) under a treatment ($do(x_{1},x_{2})$). The interventional distribution (${\mathrm{Pr}}_{x}$) is the distribution induced by the mutilated CBN $\langle G^{\prime },F^{\prime }\rangle$, where ${\mathrm{Pr}}_{x}\left(Y\right)$ corresponds to the causal effect (Pr(Y|do(x)), also notated by $\mathrm{Pr}(Y_{x})$). In this example, the causal effect of $do(x_{1},x_{2})$ on *Y* can be computed by ${\mathrm{Pr}}_{x_{1},x_{2}}\left(y\right)=\sum_{a,b,c,d,e}$$f_{A}\left(a\right)$$f_{B}\left(b,a\right)$$f_{C}\left(b,c\right)$$f_{D}\left(d,x_{1},c\right)$$f_{E}\left(e,d\right)$$f_{Y}\left(y,e,x_{2}\right)$.

### 2.2. Identifying Causal Effects

A key question in causal inference is to check whether a causal effect can be (uniquely) computed given the causal graph (*G*) and a distribution (Pr(V)) over a subset (V) of its variables. If the answer is yes, we say that the causal effect is *identifiable*, given *G* and Pr(V). Otherwise, the causal effect is *unidentifiable*. Variables (V) are said to be *observed*, and the remaining variables are said to be *hidden*, where Pr(V) is usually estimated from observational data. We start with the general definition of identifiability (not necessarily for causal effects) from [2] (Ch. 3.2.4), with a slight rephrasing.

**Definition** **1** (Identifiability [2])**.** *Let Q(M) be any computable quantity of a model (M). We say that Q is identifiable in a class of models if, for any pair of models ($M_{1}$ and $M_{2}$) from this class, $Q\left(M_{1}\right)=Q\left(M_{2}\right)$ whenever ${\mathrm{Pr}}_{M_{1}}\left(V\right)={\mathrm{Pr}}_{M_{2}}\left(V\right)$, where V represents the observed variables.*

In the context of causal effects, the problem of identifiability is to check whether every pair of fully specified CBNs ($M_{1}$ and $M_{2}$ in Definition 1) that induces the same distribution (Pr(V)) also produces the same value for the causal effect. Equivalently, to show that a causal effect is *unidentifiable*, it suffices to find two CBNs that induce the same distribution (Pr(V)) yet different causal effects. Note that Definition 1 does not restrict the considered models ($M_{1}$ and $M_{2}$) based on the properties of the distributions (${\mathrm{Pr}}_{M_{1}}\left(V\right)$ and ${\mathrm{Pr}}_{M_{2}}\left(V\right)$). However, in the literature on identifying causal effects, it is quite common to only consider CBNs (models) that induce distributions that satisfy some positivity constraints, such as Pr(V)>0. We examine such constraints more carefully in Section 3, as they may contradict functional dependencies, which we introduce later.

It is well known that under some positivity constraints (e.g., Pr(V)>0), the identifiability of causal effects can be efficiently tested using what we call the *project-ID* algorithm. Given a causal graph (*G*), project-ID first applies the projection operation proposed in [25,26,28] to yield a new causal graph ($G^{\prime }$) whose hidden variables are all roots, each with exactly two children. These properties are needed by the ID algorithm [10], which is then applied to $G^{\prime }$ to yield an identifying formula if the causal effect is identifiable and resulting in an outcome of FAIL otherwise. Consider the causal effect (${\mathrm{Pr}}_{x_{1}x_{2}}\left(y\right)$) in Figure 1a, where hidden variables are the non-root variables (B,D). We first project the causal graph (*G*) in Figure 1a onto its observed variables to yield the causal graph ($G^{\prime }$) in Figure 1c (all hidden variables in $G^{\prime }$ are auxiliary and roots). We then run the ID algorithm on $G^{\prime }$, which returns the following (simplified) identifying formula: ${\mathrm{Pr}}_{x_{1}x_{2}}\left(y\right)=$$\sum_{c}\mathrm{Pr}\left(c\right)$$\sum_{e}\mathrm{Pr}\left(y|e,x_{2}\right)$$\mathrm{Pr}(e|x_{1},c)$. Hence, the causal effect (${\mathrm{Pr}}_{x_{1}x_{2}}\left(y\right)$) is identifiable and can be computed using the above formula. Moreover, all quantities in the formula can be obtained from the distribution ($\mathrm{Pr}(A,C,E,X_{1},X_{2},Y)$) over observed variables, which can be estimated from observational data. More details on the projection operation and the ID algorithm can be found in Appendix A.

## 3. Constrained and Functional Identifiability

As mentioned earlier, Definition 1 of identifiability [2] (Ch. 3.2.4) does not restrict the pair of considered models ($M_{1}$ and $M_{2}$). However, it is common in the literature on cause–effect identifiability to only consider CBNs with distributions (Pr(V)) that satisfy some positivity constraints. Strict positivity (Pr(V)>0) is, perhaps, the mostly widely used constraint [2,9,28], that is, in Definition 1, we only consider CBNs $M_{1}$ and $M_{2}$, which induce distributions ${\mathrm{Pr}}_{M_{1}}$ and ${\mathrm{Pr}}_{M_{2}}$ that satisfy ${\mathrm{Pr}}_{M_{1}}\left(V\right)>0$ and ${\mathrm{Pr}}_{M_{2}}\left(V\right)>0$, respectively. Weaker and somewhat intricate positivity constraints were employed by the ID algorithm in [10] as discussed in Appendix A, but we apply this algorithm only under strict positivity to keep things simple (see [29,30] for a recent discussion of positivity constraints).

Positivity constraints are motivated by two considerations: technical convenience and the fact that most causal effects would be unidentifiable without some positivity constraints (more on this later). Given the multiplicity of positivity constraints considered in the literature and the subtle interaction between positivity constraints and functional dependencies (which are the main focus of this work), we next provide a systematic treatment of identifiability under positivity constraints.

### 3.1. Positivity Constraints

We first formalize the notion of a *positivity constraint*, then define the notion of *constrained identifiability*, which takes a set of positivity constraints as input (in addition to the causal graph (*G*) and distribution (Pr(V))).

**Definition** **2.** *A positivity constraint on Pr(V) is an inequality of the form Pr(S|Z)>0, where S⊆V,Z⊆V and S∩Z=∅, that is, for all instantiations (s,z), if Pr(z)>0, then Pr(s,z)>0.*

When Z=∅, the positivity constraint is defined on a marginal distribution (Pr(S)>0). To illustrate, the positivity constraint, $\mathrm{Pr}(X_{2}|X_{1},C)>0$ in Figure 1a specifies the constraint whereby $\mathrm{Pr}(x_{2},x_{1},c)>0$ if $\mathrm{Pr}(x_{1},c)>0$ for every instantiation ($x_{2},x_{1},c$). We may impose multiple positivity constraints on a set of variables (V). We use $C_{V}$ to denote the set of positivity constraints imposed on Pr(V) and $\mathrm{vars}(C_{V})$ to denote all the variables mentioned by $C_{V}$. Consider the constraints expressed as $C_{V}=\left\{\mathrm{Pr}\left(X_{1},X_{2}|B,C\right)>0,\mathrm{Pr}\left(Y|C,D\right)>0\right\}$; then, $\mathrm{vars}\left(C_{V}\right)=\left\{X_{1},X_{2},B,C,D,Y\right\}$. The weakest set of positivity constraints is $C_{V}=\left\{\right\}$ (no positivity constraints, as in Definition 1), and the strongest positivity constraint is $C_{V}=\left\{\mathrm{Pr}\left(V\right)>0\right\}$ (strict positivity).

We next provide a definition of identifiability for the causal effect of treatments (X) on outcomes (Y) in which positivity constraints are an input to the identifiability problem. We call it *constrained identifiability*, in contrast to the (unconstrained) identifiability of Definition 1.

**Definition** **3.** *We call $\langle G,V,C_{V}\rangle$ an identifiability tuple, where G is a causal graph (DAG), V is its set of observed variables, and $C_{V}$ is a set of positivity constraints.*

**Definition** **4** (Constrained Identifiability)**.** *Let $\langle G,V,C_{V}\rangle$ be an identifiability tuple. The causal effect of X on Y is said to be identifiable with respect to $\langle G,V,C_{V}\rangle$ if ${\mathrm{Pr}}_{x}^{1}\left(y\right)={\mathrm{Pr}}_{x}^{2}\left(y\right)$ for any pair of distributions (${\mathrm{Pr}}^{1}$ and ${\mathrm{Pr}}^{2}$) that are induced by G and that satisfy ${\mathrm{Pr}}^{1}\left(V\right)={\mathrm{Pr}}^{2}\left(V\right)$, as well as the positivity constraints ($C_{V}$).*

**Figure 2:**
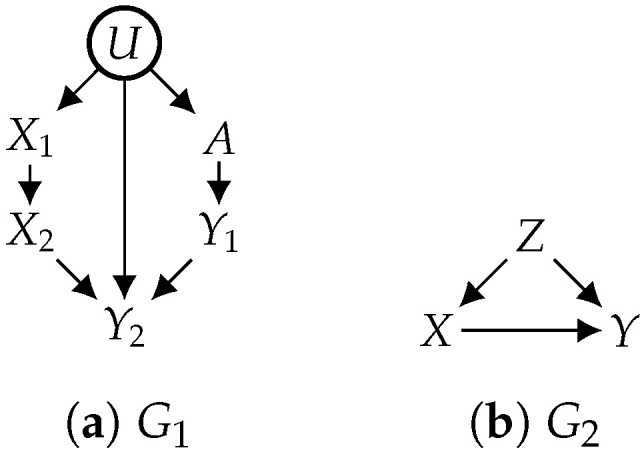
Examples for positivity.

For simplicity, we say “identifiability” to mean “constrained identifiability” in the rest of this paper. We next show that without some positivity constraints, most causal effects would not be identifiable. We first define a notion called *first ancestor* on a causal graph as follows. We say that a treatment (X∈X) is a *first ancestor* of some outcome (Y∈Y) if *X* is an ancestor of *Y* in causal graph *G* and that there exists a directed path from *X* to *Y* that is not intercepted by X∖{X}. Consider the causal graph in Figure 2a with hidden variable *U*; treatment $X_{2}$ is a first ancestor of outcome $Y_{2}$, and outcome $Y_{1}$ does not have any first ancestor. A first ancestor must exist if some treatment variable is an ancestor of some outcome variable. The following result states a criterion under which a causal effect is never identifiable.

**Proposition** **1.** *The casual effect of X on Y is not identifiable with respect to an identifiability tuple $\langle G,V,C_{V}\rangle$ if some X∈X is a first ancestor of some Y∈Y and $C_{V}$ does not imply Pr(X)>0.*

Hence, identifiability is not possible without some positivity constraints if at least one treatment variable is an ancestor of some outcome variable (which is common). According to Proposition 1, the causal effect of $\left\{X_{1},X_{2}\right\}$ on $\left\{Y_{1},Y_{2}\right\}$ is not identifiable in Figure 2a if the considered distributions do not satisfy $\mathrm{Pr}(X_{2})>0$, as $X_{2}$ is a first ancestor of $Y_{2}$.

As positivity constraints become stronger, more causal effects become more likely to be identifiable, since the set of considered models becomes smaller, that is, an unidentifiable causal effect under positivity constraint $C_{V}^{1}$ may become identifiable under positivity constraint $C_{V}^{2}$ if $C_{V}^{2}$ implies $C_{V}^{1}$. Consider the causal graph in Figure 2b, in which all variables are observed (V={X,Y,Z}). Without positivity constraints ($C_{V}=\emptyset$), the causal effect of *X* on *Y* is not identifiable. However, it becomes identifiable given strict positivity ($C_{V}=\left\{\mathrm{Pr}\left(X,Y,Z\right)>0\right\}$), leading to an identifying formula expressed as ${\mathrm{Pr}}_{x}\left(y\right)=\sum_{z}\mathrm{Pr}\left(y|x,z\right)\mathrm{Pr}\left(z\right)$. This causal effect is also identifiable under the weaker positivity constraint, i.e., $C_{V}=\left\{\mathrm{Pr}\left(X|Z\right)>0\right\}.$ In this example, the positivity assumption ($C_{V}=\left\{\mathrm{Pr}\left(X,Y,Z\right)>0\right\}$) is sufficient to make the identifying formula well defined because Pr(y|x,z)Pr(z) in the formula is equal to zero when Pr(z)=0 and is computable when Pr(z)>0 (the conditional probability Pr(y|x,z) is well defined if Pr(x|z)>0). This is an example where strict positivity may be assumed for technical convenience only, as it may facilitate the application of some identifiability techniques like do-calculus [2].

### 3.2. Functional Dependencies

A variable (*X*) in a causal graph is said to *functionally depend* on its parents (P) if its distribution is deterministic (Pr(x|p)∈{0,1}) for every instantiation (x,p). Variable *X* is also said to be *functional* in this case. In this work, we assume *qualitative* functional dependencies. *We do not know the distribution (Pr(X|P)); we only know that it is deterministic.* We assume that root variables cannot be functional, as such variables can be removed from the causal graph.

The table on the right shows two variables (*B* and *C*) that both have *A* as their parent. Variable *C* is functional, but variable *B* is not. The CPT for variable *C* is called a *functional CPT* in this case. Functional CPTs are also known as (causal) mechanisms and are expressed using structural equations in structural causal models (SCMs) [31,32,33]. By definition, in an SCM, every non-root variable is assumed to be functional (when noise variables are represented explicitly in the causal graph).
*A**B**C*Pr(B|A)Pr(C|A)0000.200110.811000.611110.40

Qualitative functional dependencies are a longstanding concept. For example, they are common in relational databases (see, e.g., [34,35]), and their relevance to probabilistic reasoning was previously brought up in [20] (Ch. 3). One example of a (qualitative) functional dependency is that different countries have different driving ages, so we know that “driving age” functionally depends on “country”, even though we may not know the specific driving age for each country. Another example is that a “Letter grade” for a class is functionally dependent on the student’s “weighted average”, even though we may not know the scheme for converting a weighted average to a letter grade.

In this work, we assume that we are given a causal graph (*G*) in which some variables (W) have been designated as functional. The presence of functional variables further restricts the set of distributions (Pr) that we consider when checking identifiability. This leads to a more refined problem that we call *functional identifiability (F-identifiability)*, which depends on four elements.

**Definition** **5.** *We call $\langle G,V,C_{V},W\rangle$ an F-identifiability tuple when G is a DAG, V is its set of observed variables, $C_{V}$ is a set of positivity constraints, and W is a set of functional variables in G.*

**Definition** **6** (F-Identifiability)**.** *Let $\langle G,V,C_{V},W\rangle$ be an F-identifiability tuple. The causal effect of X on Y is F-identifiable with respect to $\langle G,V,C_{V},W\rangle$ if ${\mathrm{Pr}}_{x}^{1}\left(y\right)={\mathrm{Pr}}_{x}^{2}\left(y\right)$ for any pair of distributions (${\mathrm{Pr}}^{1}$ and ${\mathrm{Pr}}^{2}$) that are induced by G, that satisfy ${\mathrm{Pr}}^{1}\left(V\right)={\mathrm{Pr}}^{2}\left(V\right)$ and the positivity constraints ($C_{V}$), and in which variables (W) functionally depend on their parents.*

Both $C_{V}$ and W represent constraints on the models (CBNs) we consider when checking identifiability, and these two types of constraints may contradict each other. We next define two notions that characterize some important interactions between positivity constraints and functional variables.

**Definition** **7.** *Let $\langle G,V,C_{V},W\rangle$ be an F-identifiability tuple. Then, $C_{V}$ and W are consistent if there exists a parameterization for G that induces a distribution satisfying $C_{V}$ and in which variables (W) functionally depend on their parents. Moreover, $C_{V}$ and W are separable if $W\cap \mathrm{vars}(C_{V})=\emptyset$.*

If $C_{V}$ is inconsistent with W, then the set of distributions (Pr) considered in Definition 6 is empty; hence, the causal effect is not well defined (and trivially identifiable according to Definition 6). As such, one would usually want to ensure such consistency. Here are some examples of positivity constraints that are always consistent with a set of functional variables (W): positivity foreach treatment variable, i.e., {Pr(X)>0,X∈X}; positivity for the set of non-functional treatments, i.e., {Pr(X∖W)>0}; and positivity for all non-functional variables, i.e., {Pr(V∖W)>0}. It turns out that all these examples are special cases of the following condition. For a functional variable (W∈W), let $H_{W}$ be variables that intercept all directed paths from non-functional variables to *W* (such a $H_{W}$ may not be unique). If none of the positivity constraints in $C_{V}$ mentions both *W* and $H_{W}$, then $C_{V}$ and W are guaranteed to be consistent (see Proposition A4 in Appendix C).

Separability is a stronger condition, and it intuitively implies that the positivity constraints do not rule out any possible functions for the variables in W. We need such a condition for one of the results we present later. Some examples of positivity constraints that are separable from W are {Pr(X∖W)>0} and {Pr(V∖W)>0}. Studying the interactions between positivity constraints and functional variables, as we do in this section, will prove helpful later when utilizing existing identifiability algorithms (which require positivity constraints) for the testing of functional identifiability.

## 4. Functional Elimination and Projection

Our approach for testing identifiability under functional dependencies is based the elimination of functional variables from the causal graph, followed by the invocation of the project-ID algorithm on the resulting graph. This can be subtle, though, since the described process does not work for every functional variable, as we discuss in the next section. Moreover, one needs to handle the interaction between positivity constraints and functional variables carefully. However, the first step is to formalize the process of eliminating a functional variable and to study the associated guarantees.

Eliminating variables from a probabilistic model is a well studied operation also known as marginalization (see, e.g., [36,37,38]). When eliminating variable *X* from a model that represents distribution Pr(Z), the goal is to obtain a model that represents the marginal distribution ($\mathrm{Pr}\left(Y\right)=\sum_{x}\mathrm{Pr}\left(x,Y\right)$, where Y=Z∖{X}). Elimination can also be applied to a DAG (*G*) that represents conditional independencies (I), leading to a new DAG ($G^{\prime }$) that represents independencies ($I^{\prime }$) that are implied by I. In fact, the projection operation we discussed earlier [25,26] can be understood in these terms. We next propose an operation that eliminates functional variables from a DAG and that comes with stronger guarantees compared to earlier elimination operations as far as preserving independencies.

**Definition** **8.** *The functional elimination of a variable (X) from a DAG (G) yields a new DAG attained by adding an edge from each parent of X to each child of X, then removing X from G.*

**Figure 3:**
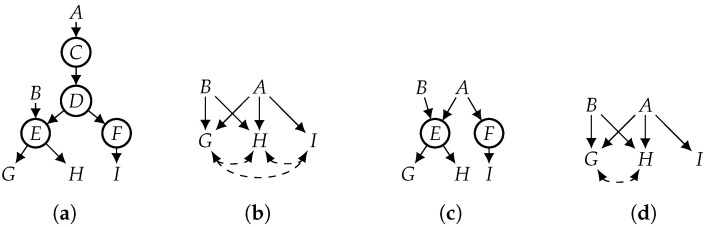
Contrasting projection with functional projection. *C* and *D* are functional. Hidden variables are circled. (**a**) DAG; (**b**) proj. (**a**) on *A*, *B*, *G*, *H*, *I*; (**c**) eliminate C,D from (**a**); (**d**) proj. (**c**) on *A*, *B*, *G*, *H*, *I*.

Appendix B extends this definition to causal Bayesian networks (i.e., updating both CPTs and the causal graph). For convenience, we sometimes say “elimination” to mean “functional elimination” when the context is clear. From the viewpoint of independence relations, functional elimination is not sound if the eliminated variable is not functional. In particular, the DAG ($G^{\prime }$) that results from this elimination process may satisfy independencies (identified by d-separation) that do not hold in the original DAG (*G*). As we show later, however, every independence implied by $G^{\prime }$ must be implied by *G* if the eliminated variable is functional. In the context of SCMs, functional elimination may be interpreted as replacing the eliminated variable (*X*) with its function in all structural equations that contain *X*. Functional elimination applies in broader contexts than SCMs, though. Eliminating multiple functional variables in any order yields the same DAG (see Proposition A3 in Appendix B). For example, eliminating variables {C,D} from the DAG in Figure 3a yields the DAG in Figure 3c whether we use the order of $\pi_{1}=C,D$ or the order of $\pi_{2}=D,C$.

Functional elimination preserves independencies that hold in the original DAG and that are not preserved by other elimination methods, including projection, as defined in [25,26]. These independencies are captured using the notion of D-separation [39,40], which is more refined than the classical notion of d-separation [41,42] (uppercase D versus lowercase d). The original definition of D-separation can be found in [40]. We provide a simpler definition next, stated as Proposition 2, as the equivalence between the two definitions is not immediate.

**Proposition** **2.** *Let X,Y,Z be disjoint variable sets and W be a set of functional variables in DAG G. Then, X and Y are D-separated by Z in 〈G,W〉 iff X and Y are d-separated by $Z^{\prime }$ in G, where $Z^{\prime }$ is obtained as follows. Initially, $Z^{\prime }=Z$. The next step is repeated until $Z^{\prime }$ stops changing. Then, every variable in W whose parents are in $Z^{\prime }$ is added to $Z^{\prime }$.*

To illustrate the difference between d-separation and D-separation, consider, again, the DAG in Figure 3a and assume that variables *C* and *D* are functional. Variables *G* and *I* are not d-separated by *A*, but they are D-separated by *A*, that is, there are distributions that are induced by the DAG in Figure 3a and in which *G* and *I* are not independent given *A*. However, *G* and *I* are independent given *A* in every induced distribution in which variables *C* and *D* are functionally determined by their parents. Functional elimination preserves D-separation in the following sense.

**Theorem** **1.** *Consider a DAG (G) with functional variables (W). Let $G^{\prime }$ be the result of functionally eliminating variables $W^{\prime }\subseteq W$ from G. For any disjoint sets (X, Y, and Z) in $G^{\prime }$, X and Y are D-separated by Z in 〈G,W〉 iff X and Y are D-separated by Z in $\langle G^{\prime },W\setminus W^{\prime }\rangle$.*

The above result is stated with respect to eliminating a subset of the functional variables. If we eliminate all functional variables, then D-separation is reduced to d-separation. For example, variables *G* and *I* are D-separated by *A* in Figure 3c and in Figure 3a as suggested by Theorem 1. In fact, *G* and *I* are also d-separated by *A* in Figure 3c, since we eliminated all functional variables. We now have the following stronger result.

**Corollary** **1.** *Consider a DAG (G) with functional variables (W). Let $G^{\prime }$ be the result of functionally eliminating all variables (W) from G. For any disjoint sets (X, Y, and Z) in $G^{\prime }$, X and Y are d-separated by Z in $G^{\prime }$ iff X and Y are D-separated by Z in 〈G,W〉.*

We now define the operation of functional projection, which augments the original projection operation proposed in [25,26] in the presence of functional dependencies.

**Definition** **9.** *Let G be a DAG, V be its observed variables, and W be its hidden functional variables (W∩V=∅). The functional projection of G on V is a DAG obtained by functionally eliminating variables (W) from G, then projecting the resulting DAG on variables (V).*

We now contrast functional projection and classical projection using the causal graph in Figure 3a, assuming that the observed variables are V={A,B,G,H,I} and the functional variables are W={C,D}. Applying classical projection to this causal graph yields the causal graph in Figure 3b. To apply functional projection, we first functionally eliminate *C* and *D* from Figure 3a, which yields Figure 3c; then, we project Figure 3c on variables (V), which yields the causal graph in Figure 3d. So we now need to contrast Figure 3b (classical projection) with Figure 3d (functional projection). The latter is a strict subset of the former, as it is missing two bidirected edges. One implication of this is that variables *G* and *I* are not d-separated by *A* in Figure 3b because they are not d-separated in Figure 3a. However, they are D-separated in Figure 3a; hence, they are d-separated in Figure 3d. So functional projection yields a DAG that exhibits more independencies. Again, this is because *G* and *I* are D-separated by *A* in the original DAG, a fact that is not visible to the projection but is visible to (and exploitable by) the functional projection.

An important corollary of functional projection is the following.

**Corollary** **2.** *Let G be a DAG; V be its observed variables; W be its functional variables, which are all hidden; and $G^{\prime }$ be the result of functionally projecting G on V. For any disjoint sets (X, Y, and Z) in $G^{\prime }$, X and Y are d-separated by Z in $G^{\prime }$ iff X and Y are D-separated by Z in 〈G,W〉.*

In other words, classical projection preserves d-separation, but functional projection preserves D-separation, which subsumes d-separation. Corollary 2 is a bit more subtle and powerful than it may first seem. First, it concerns D-separations based on hidden functional variables, not all functional variables. Secondly, it shows that such D-separations in *G* appear as classical d-separations in $G^{\prime }$ which allows us to feed $G^{\prime }$ into existing identifiability algorithms, as we show later. This is a key enabler of some results we present next on the testing of functional identifiability.

## 5. Causal Identification with Functional Dependencies

**Figure 4:**
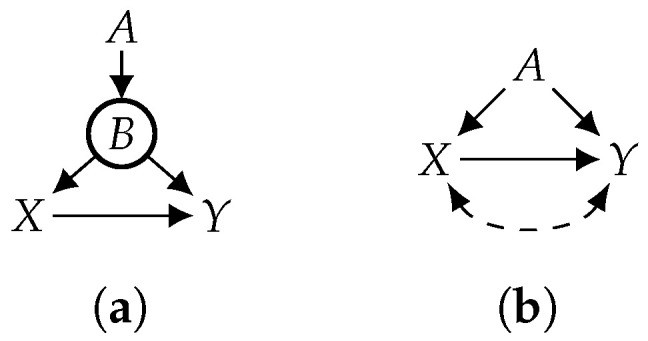
*B* is functional. (**a**) DAG; (**b**) projection.

Consider the causal graph (*G*) in Figure 4a and let V={A,X,Y} be its observed variables. According to Definition 4 of identifiability, the causal effect of *X* on *Y* is not identifiable with respect to $\langle G,V,C_{V}\rangle$, where $C_{V}=\left\{\mathrm{Pr}\left(A,X,Y\right)>0\right\}$. We can show this by projecting the causal graph (*G*) on the observed variables (V), which yields the causal graph ($G^{\prime }$) in Figure 4b, then applying the ID algorithm to $G^{\prime }$, which returns FAIL. Suppose now that the hidden variable (*B*) is known to be functional. According to Definition 6 of F-identifiability, this additional knowledge reduces the number of considered models, so it actually renders the causal effect identifiable—the identifying formula is ${\mathrm{Pr}}_{x}\left(y\right)=\sum_{a}$Pr(a)Pr(y|a,x), as we show later. Hence, an unidentifiable causal effect becomes identifiable in light of knowledge that some variable is functional, even without knowing the structural equations for this variable.

The question now is how to algorithmically test F-identifiability. We propose two techniques for this purpose, the first of which is geared towards exploiting existing algorithms for classical identifiability. This technique is based on the elimination of functional variables from the causal graph while preserving F-identifiability, with the goal of getting to a point where F-identifiability becomes equivalent to classical identifiability. If we reach this point, we can use existing algorithms for classical identifiability, like the ID algorithm, to test F-identifiability. This can be subtle, though, since hidden functional variables behave differently from observed ones. We start with the following result.

**Theorem** **2.** *Let $\langle G,V,C_{V},W\rangle$ be an F-identifiability tuple. If $G^{\prime }$ is the result of functionally eliminating the hidden functional variables (W∖V) from G, then the causal effect of X on Y is F-identifiable with respect to $\langle G,V,C_{V},W\rangle$ iff it is F-identifiable with respect to $\langle G^{\prime },V,C_{V},V\cap W\rangle$.*

An immediate corollary of this theorem is that if all functional variables are hidden, then we can reduce the question of F-identifiability to identifiability, since V∩W=∅, so F-identifiability with respect to $\langle G^{\prime },V,C_{V},V\cap W=\emptyset \rangle$ collapses into identifiability with respect to $\langle G^{\prime },V,C_{V}\rangle$.

**Corollary** **3.** *Let $\langle G,V,C_{V},W\rangle$ be an F-identifiability tuple, where $C_{V}=\left\{\mathrm{Pr}\left(V\right)>0\right\}$ and W are all hidden. If $G^{\prime }$ is the result of functionally projecting G on variables (V), then the causal effect of X on Y is F-identifiable with respect to $\langle G,V,C_{V},W\rangle$ iff it is identifiable with respect to $\langle G,V,C_{V}\rangle$ (We require the positivity constraint Pr(V)>0, as we suspect the projection operation in [26] requires it even though that was not made explicit in the paper; if not, then $C_{V}$ can be empty in Corollary 3).*

**Figure 5:**
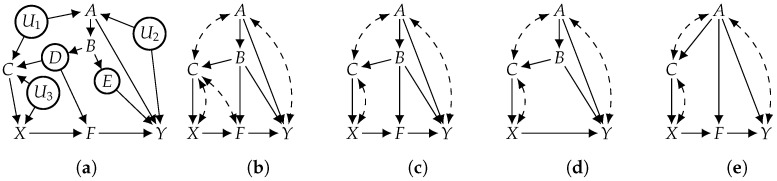
Variables A,B,C,F,X, and *Y* are observed. Variables *D* and *E* are functional (and hidden). (**a**) Causal graph; (**b**) proj. of (**a**); (**c**) F-proj. of (**a**); (**d**) F-elim. *F*; (**e**) F-elim. *B*.

This corollary suggests a method for using the ID algorithm, which is popular for testing identifiability, to establish F-identifiability by coupling ID with functional projection instead of classical projection. Consider the causal graph (*G*) in Figure 5a with observed variables of V={A,B,C,F,X,Y}. The causal effect of *X* on *Y* is not identifiable under Pr(V)>0; projecting *G* on observed variables (V) yields the causal graph ($G^{\prime }$) in Figure 5b, and the ID algorithm produces FAIL on $G^{\prime }$. Suppose now that the hidden variables ({D,E}) are functional. To test whether the causal effect is F-identifiable using Corollary 3, we functionally project *G* on the observed variables (V), which yields the causal graph ($G^{\prime \prime }$) in Figure 5c. Applying the ID algorithm to $G^{\prime \prime }$ produces the following identifying formula: ${\mathrm{Pr}}_{x}\left(y\right)=\sum_{bf}\mathrm{Pr}\left(f|b,x\right)\sum_{acx^{\prime }}\mathrm{Pr}\left(y|a,b,c,f,x^{\prime }\right)\mathrm{Pr}\left(a,b,c,x^{\prime }\right)$; therefore, ${\mathrm{Pr}}_{x}\left(y\right)$ is F-identifiable.

We stress, again, that Corollary 3 and the corresponding F-identifiability algorithm apply only when all functional variables are hidden. We now treat the case when some of the functional variables are observed. The subtlety here is that, unlike hidden functional variables, eliminating an observed functional variable does not always preserve F-identifiability. However, the following result identifies conditions that guarantee the preservation of F-identifiability based on the notion of separability in Definition 7. If all observed functional variables satisfy these conditions, we can, again, reduce F-identifiability into identifiability, so we can exploit existing methods for identifiability like the ID algorithm and do-calculus.

**Theorem** **3.** *Let $\langle G,V,C_{V},W\rangle$ be an F-identifiability tuple. Let Z be a set of observed functional variables that are neither treatments nor outcomes, are separable from $C_{V}$, and have observed parents. If $G^{\prime }$ is the result of functionally eliminating variables (Z) from G, then the causal effect of X on Y is F-identifiable with respect to $\langle G,V,C_{V},W\rangle$ iff it is F-identifiable with respect to $\langle G^{\prime },V\setminus Z,C_{V},W\setminus Z\rangle$.*

Intuitively, the theorem allows us to remove observed functional variables from a causal graph if they satisfy the given conditions. We now have the following important corollary of Theorems 2 and 3, which subsumes Corollary 3.

**Corollary** **4.** *Let $\langle G,V,C_{V},W\rangle$ be an F-identifiability tuple, where $C_{V}=\left\{\mathrm{Pr}\left(V\setminus W\right)>0\right\}$ and every variable in W∩V satisfies the conditions of Theorem 3. If $G^{\prime }$ is the result of functionally projecting G on V∖W, then the causal effect of X on Y is F-identifiable with respect to $\langle G,V,C_{V},W\rangle$ iff it is identifiable with respect to $\langle G^{\prime },V\setminus W,C_{V}\rangle$.*

Consider, again, the causal effect of *X* on *Y* in graph *G* of Figure 5a with observed variables of V={A,B,C,F,X,Y}. Suppose now that the observed variable (*F*) is also functional (in addition to hidden functional variables *D* and *E*) and assume Pr(A,B,C,X,Y)>0. Using Corollary 4, we can functionally project *G* on *A*, *B*, *C*, *X*, and *Y* to yield the causal graph ($G^{\prime }$) in Figure 5d, which reduces F-identifiability on *G* to classical identifiability on $G^{\prime }$. Since strict positivity holds in $G^{\prime }$, we can apply any existing identifiability algorithm and conclude that the causal effect is not identifiable. For another scenario, suppose that the observed variable (*B*) (instead of *F*) is functional and we have Pr(A,C,F,X,Y)>0. Again, using Corollary 4, we functionally project *G* onto *A*, *C*, *F*, *X*, and *Y* to yield the causal graph ($G^{\prime \prime }$) in Figure 5e, which reduces F-identifiability on *G* to classical identifiability on $G^{\prime \prime }$. If we apply the ID algorithm to $G^{\prime \prime }$, we obtain the following identifying formula (which we denote as Equation (A1)): ${\mathrm{Pr}}_{x}\left(y\right)=\sum_{af}\mathrm{Pr}\left(f|a,x\right)\sum_{cx^{\prime }}\mathrm{Pr}\left(y|a,c,f,x^{\prime }\right)\mathrm{Pr}\left(a,c,x^{\prime }\right)$. In both scenarios presented above, we were able to test F-identifiability using an existing algorithm for identifiability.

Corollary 4 (and Theorem 3) has yet another key application: it can help us pinpoint observations that are not essential for identifiability. To illustrate, consider the second scenario presented above, where the observed variable (*B*) is functional in the causal graph (*G*) of Figure 5a. The fact that Corollary 4 allowed us to eliminate variable *B* from *G* implies that observation of this variable is not needed to render the causal effect F-identifiable and, hence, is not needed to compute the causal effect. This can be seen by examining the identifying formula (Equation (A1)), which does not contain variable *B*. This can be further generalized to the causal graph on the right with functional variables ($B_{1},\cdots ,B_{n}$), where we assume Pr(A,C,X,D,Y)>0. According to Corollary 4, we can functionally project the graph onto $V^{\prime }=\left\{A,C,X,D,Y\right\}$ while preserving F-identifiability. Moreover, applying the ID algorithm (or do-calculus) to *G* yields an identifying formula for ${\mathrm{Pr}}_{x}\left(Y\right)$ over only $V^{\prime }$, that is, in this example, we only need to observe a constant number (five) of variables to render the causal effect F-identifiable, even though the number of observed variables in the original graph is unbounded. This application of Corollary 4 can be quite significant in practice, especially when some variables are expensive to measure (observe) or when they may raise privacy concerns see (e.g., [43,44]).

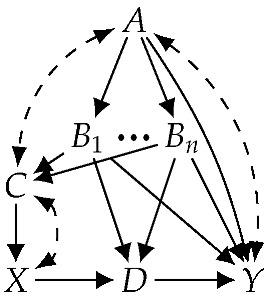



Theorems 2 and 3 are more far-reaching than what the above discussion may suggest. In particular, even if we cannot eliminate every (observed) functional variable using these theorems, we may still be able to reduce F-identifiability to identifiability due to the following result.

**Theorem** **4.** *Let $\langle G,V,C_{V},W\rangle$ be an F-identifiability tuple. If every functional variable has at least one hidden parent, then a causal effect of X on Y is F-identifiable with respect to $\langle G,V,C_{V},W\rangle$ iff it is identifiable with respect to $\langle G,V,C_{V}\rangle$.*

That is, if we still have functional variables in the causal graph after applying Theorems 2 and 3 and if each such variable has at least one hidden parent, then F-identifiability is equivalent to identifiability. Consider, again, the causal effect of *X* on *Y* in *G* of Figure 5a with observed variables of V={A,B,C,F,X,Y}. Now, suppose that the observed variables (*A*, *B*, *C*, *X*, and *Y* are also functional (in addition to hidden functional variables *D* and *E*) and assume Pr(A,C,F,X,Y)>0. We can reduce F-identifiability to classical identifiability by combining Theorems 3 and 4. In particular, according to Theorem 3, we first reduce F-identifiability on *G* to F-identifiability on $G^{\prime }$ in Figure 5e by functionally eliminating *D*, *E*, and *B*. Since all the remaining functional variables (*A*, *C*, *X*, and *Y*) have a hidden parent, we can further reduce F-identifiability to identifiability on $G^{\prime }$ according to Theorem 4, then apply existing algorithms (e.g., ID and do-calculus) to conclude that the causal effect is identifiable.

The method we have presented thus far for the testing of F-identifiability is based on the elimination of functional variables from the causal graph, followed by the application of existing tools for causal effect identification, such as the project-ID algorithm and do-calculus. This F-identifiability method is complete if every observed functional variable either satisfies the conditions of Theorem 3 or has at least one hidden parent that is not functional.

We next present another technique for reducing F-identifiability to identifiability. This method is more general and much more direct than the previous one, but it does not allow us to fully exploit some existing tools, like the ID algorithm, due to the positivity assumptions they make. The new method involves pretending that some of the hidden functional variables are actually observed, inspired by Proposition 2, which reduces D-separation to d-separation using a similar technique.

**Theorem** **5.** *Let $\langle G,V,C_{V},W\rangle$ be an F-identifiability tuple, where $C_{V}=\left\{\mathrm{Pr}\left(X\right)>0,X\in X\right\}$. A causal effect of X on Y is F-identifiable with respect to $\langle G,V,C_{V},W\rangle$ iff it is identifiable with respect to $\langle G,C_{V},V^{\prime }\rangle$, where $V^{\prime }$ is obtained as follows. Initially, $V^{\prime }=V$. This is repeated until $V^{\prime }$ stops changing. Then, a functional variable from W is added to $V^{\prime }$ if its parents are in $V^{\prime }$.*

Consider the causal effect of *X* on *Y* in graph *G* of Figure 5a and suppose the observed variables are V={A,B,C,X,Y}; the functional variables are *D*, *E*, and *F*; and we have Pr(X)>0. According to Theorem 5, the causal effect of *X* on *Y* is F-identifiable iff it is identifiable in *G* while pretending that variables $V^{\prime }=\{A$, *B*, *C*, *D*, *E*, *F*, *X*, Y} are all observed. In this case, the casual effect is not identifiable, but we cannot obtain this answer by applying an identifiability algorithm that requires positivity constraints that are stronger than Pr(X)>0. If we have stronger positivity constraints that imply Pr(X)>0,X∈X, then only the if part of Theorem 5 holds, assuming $C_{V}$ and W are consistent, that is, confirming identifiability with respect to $\langle G,C_{V},V^{\prime }\rangle$ confirms F-identifiability with respect to $\langle G,V,C_{V},W\rangle$, but if identifiability is not confirmed, then F-identifiability may still hold. This suggests that to fully exploit the power of Theorem 5, one would need a new class of identifiability algorithms that can operate under the weakest possible positivity constraints.

## 6. Experiments

We next report on a simple experiment to empirically demonstrate how knowledge of functional dependencies can aid the identifiability of causal effects. We randomly generated 50 causal graphs (DAGs) with N∈{50,100,150} variables using the Erdős–Rényi method [45], where every edge in the causal graphs appears with a probability of 0.1 and every variable has, at most, 6 parents. We then randomly picked 0.8N observed variables, 0.2N treatment variables, 0.2N outcome variables, and W∈{0.25N,0.5N,0.75N} functional variables from the causal graphs.

For each combination of *N* and *W*, Table 1 records the number of causal effects (out of 50) that are (1) unidentifiable (uid); and (2) unidentifiable but F-identifiable (uid-fid). The table also records the average number of observed variables after applying Theorems 2–4 (#obs); these are the observed variables passed to the project-ID algorithm (We assume that strict positivity holds for the remaining observed variables after applying Theorems 2–4).

The following patterns are clear. First, more unidentifiable causal effects become F-identifiable when more variables exhibit functional dependencies. This observation demonstrates that knowledge of functional dependencies can greatly improve the identifiability of causal effects. Secondly, the number of observed variables required by the project-ID algorithm becomes smaller when there are more functional variables, implying that we only need to collect data on a smaller set of variables to estimate the (identifiable) causal effects. Again, this is because more observed functional variables can be functionally eliminated from the causal graphs by Theorem 3.

## 7. Conclusions

We studied the identification of causal effects in the presence of a particular type of knowledge called functional dependencies. This augments earlier works that considered other types of knowledge, such as context-specific independence. Our contributions include the formalization of the notion of functional identifiability; the introduction of an operation for eliminating functional variables from a causal graph that comes with stronger guarantees compared to earlier elimination methods; and the employment (under some conditions) of existing algorithms, such as the ID algorithm, for the testing of functional identifiability and to obtain identifying formulas. We further provided a complete reduction of functional identifiability to classical identifiability under very weak positivity constraints and showed how our results can be used to reduce the number of variables needed in observational data. Last but not least, we proposed a more general definition of identifiability based on a broader class of positivity assumptions, which opens the door to uncover causal identification algorithms under weaker positivity assumptions.

## Figures and Tables

**Table 1:** Numbers of causal effects that are unidentifiable (uid) and that are unidentifiable but F-identifiable (uid-fid) and average number of observed variables passed to project-ID (#obs) for causal graphs with various numbers of variables (*N*) and functional ones (*W*).

*N*	*W*	uid	uid-fid	#obs
50	0.25N	15	3	36.5
	0.5N	15	10	32.6
	0.75N	15	11	27.3
100	0.25N	50	0	74.7
	0.5N	50	6	68.1
	0.75N	50	18	57.3
150	0.25N	50	0	113.2
	0.5N	50	2	103.6
	0.75N	50	5	89.7

## Data Availability

Data are contained within the article.

## Appendix A. More on Projection and the ID Algorithm

As mentioned in the main paper, the project-ID algorithm involves two steps: the projection operation and the ID algorithm. We review more technical details of each step in this section.

### Appendix A.1. Projection

The projection [25,26,28] of *G* onto V constructs a new DAG ($G^{\prime }$) over variables (V) as follows. Initially, DAG $G^{\prime }$ contains variables (V) but no edges. Then, for every pair of variables (X,Y∈V), an edge is added from *X* to *Y* to $G^{\prime }$ if *X* is a parent of *Y* in *G* or if there exists a directed path from *X* to *Y* in *G* such that none of the internal nodes on the path is in V. Furthermore, a bidirected edge (X⤎⤏Y) is added between every pair of variables (*X* and *Y*) in $G^{\prime }$ if there exists a divergent path (A divergent path between *X* and *Y* is a path in the form of X←⋯←U→⋯→Y) between *X* and *Y* in *G* such that none of the internal nodes on the path is in V. For example, the projection of the DAG in Figure 1a onto $A,C,E,X_{1},X_{2},Y$ yields Figure 1c. A bidirected edge (X⤎⤏Y) is a compact notation for X←H→Y, where *H* is an auxiliary hidden variable. Hence, the projected DAG in Figure 1c can be interpreted as a classical DAG but with additional, hidden root variables.

The projection operation is guaranteed to produce a DAG ($G^{\prime }$) in which hidden variables are all roots and each has exactly two children. Graphs that satisfy this property are called *semi-Markovian* and can be fed as inputs to the ID algorithm for testing of identifiability [10]. Moreover, projection preserves some properties of *G*, such as d-separation [25] among V, which guarantees that identifiability is preserved when working with $G^{\prime }$ instead of *G* [26].

### Appendix A.2. ID Algorithm

After obtaining a projected causal graph, we can apply the ID algorithm for identification of causal effects [10,46]. The algorithm returns either an identifying formula if the causal effect is identifiable or FAIL otherwise. The algorithm is sound, since each line of the algorithm can be proven with basic probability rules and do-calculus. The algorithm is also complete, since a causal graph must contain a *hedge*, a graphical structure that induces the unidentifiability, if the algorithm returns FAIL. However, the algorithm is only sound and complete under certain positivity constraints, which are weaker but more subtle than strict positivity (Pr(V)>0).

The positivity constraints required by ID can be summarized as follows (X represents the treatment variables): (1) Pr(X|P)>0, where P=Parents(X)∖X, and (2) Pr(Z)>0 for all quantities Pr(S|Z) considered by the ID algorithm. The second constraint depends on a particular run of the ID algorithm and can be interpreted as follows. First, if the ID algorithm returns FAIL, then the causal effect is not identifiable, even under the strict positivity constraint of Pr(V)>0. However, if the ID algorithm returns “identifiable”, then the causal effect is identifiable under the above constraints, which are now well defined given a particular run of the ID algorithm. We illustrate with an example next.

Consider the causal graph on the right, which contains observed variables {A,B,C,X,Y}. Suppose we are interested in the causal effect of *X* on *Y*. Applying the ID algorithm returns the following identifying formula: ${\mathrm{Pr}}_{x}\left(y\right)$$=\sum_{abc}$Pr(c|a,b,x)$\sum_{x^{\prime }}\mathrm{Pr}\left(y|a,b,c,x^{\prime }\right)$$\mathrm{Pr}(a,b,x^{\prime })$. The positivity constraint extracted from this run of the algorithm is Pr(a,b,c,x)>0 for all of *a*, *b*, *c*, and *x*, that is, we can only safely declare the causal effect identifiable based on the ID algorithm if this positivity constraint is satisfied.

## Appendix B. Functional Elimination for CBNs

The functional elimination in Definition 8 removes functional variables from a DAG (*G*) and yields another DAG ($G^{\prime }$) for the remaining variables. We showed in the main paper that the functional elimination preserves the D-separations. Here, we extend the notion of functional elimination to causal Bayesian networks (CBNs), which contain not only a causal graph (DAG) but also CPTs. We show that (extended) functional elimination preserves the marginal distribution of the remaining variables, that is, given any CBN with causal graph *G*, we can construct another CBN with causal graph $G^{\prime }$ such that the two CBNs induce the same distribution of the variables in $G^{\prime }$, where $G^{\prime }$ is the result of eliminating functional variables from *G*. Moreover, we show that the functional elimination operation further preserves the causal effects, which makes it applicable to causal identification. This extended version of functional elimination and the corresponding results are used as proofs in Appendix C.

Recall that a CBN 〈G,F〉 contains a causal graph (*G*) and a set of CPTs (F). We first extend the definition of functional elimination (Definition 8) from DAGs to CBNs.

**Definition** **A1.**
*The functional elimination of a functional variable (X) from a CBN 〈G,F〉 yields another CBN $\langle G^{\prime },F^{\prime }\rangle$ obtained as follows. The DAG ($G^{\prime }$) is obtained from G according to Definition 8. For each child (C) of X, its CPT in $F^{\prime }$ is $\left(\sum_{X}f_{X}f_{C}\right)$, where $f_{X}$ and $f_{C}$ are the corresponding CPTs in F.*

We first show that the new CPTs produced by Definition A1 are well defined.

**Proposition** **A1.**
*Let $f_{X}$ and $f_{Y}$ be the CPTs for variables X and Y in a CBN; then, $\left(\sum_{X}f_{X}f_{Y}\right)$ is a valid CPT for Y.*

The next proposition shows that functional elimination preserves the functional dependencies.

**Proposition** **A2.**
*Let $M^{\prime }$ be the CBN resulting from functionally eliminating a functional variable from a CBN (M). Then, each variable (from $M^{\prime }$) is functional in $M^{\prime }$ if it is functional in M.*

The next theorem shows that the order of functional elimination does not matter.

**Proposition** **A3.**
*Let M be a CBN and $\pi_{1}$ and $\pi_{2}$ be two variable orders over a set of functional variables (W). Then, functionally eliminating W from M according to $\pi_{1}$ and $\pi_{2}$ yields the same CBN.*

The next result shows that eliminating functional variables preserves the marginal distribution.

**Theorem** **A1.** *Consider a CBN (M) that induces* Pr*. Let $M^{\prime }$ be the result of functionally eliminating a set of functional variables (W) from M, which induces ${\mathrm{Pr}}^{\prime }$. Then, ${\mathrm{Pr}}^{\prime }=\sum_{W}\mathrm{Pr}$.*

One key property of functional elimination is that it preserves the interventional distribution over the remaining variables. This property allows us to eliminate functional variables from a causal graph and estimate the causal effects in the resulting graph.

**Theorem** **A2.**
*Let $M^{\prime }$ be the CBN over variables (V) resulting from functionally eliminating a set of functional variables (W) from a CBN (M). Then, $M^{\prime }$ and M attain the same ${\mathrm{Pr}}_{x}\left(V\right)$ for any X⊆V.*

## Appendix C. Proofs

The proofs of the results are ordered slightly differently from the order in which they appear in the main body of the paper.

**Proof of Proposition** **1.** Our goal is to construct two different parameterizations ($F^{1}$ and $F^{2}$) that induce the same Pr(V) but different ${\mathrm{Pr}}_{x}\left(y\right)$. This is done by first creating a parameterization (F) that contains strictly positive CPTs for all variables, then constructing $F^{1}$ and $F^{2}$ based on F. Let P be the directed path from $X=X_{i}$ to *Y*, denoted as X→Z→⋯→Y, which does not contain any treatment variables other than *X*. Let $P_{X}$ be the parents of *X* in *G*. For each node *M* on the path, let $P_{M}$ be the parents of *M*, except for the parent that lies on P. Moreover, for each variable (*M*) on P, we only modify the conditional probability for a single state ($m^{*}$) of *M*, where $x^{*}\in x$ is the treated state of *X*. Let ϵ be an arbitrarily small constant (close to 0). Next, we show the modifications for the CPTs in $F^{1}$.

$$f^{1}\left(x|p_{X}\right)=\left\{\begin{array}{cc} 0 & \mathrm{if}\;x=x^{*}\\ 1/(|X|-1), & \mathrm{otherwise}\; \end{array}\right.$$

$$f^{1}\left(z|x,p_{Z}\right)=\left\{\begin{array}{cc} 1-\epsilon , & \mathrm{if}\;x=x^{*},z=z^{*}\\ \epsilon /(|Z|-1), & \mathrm{if}\;x=x^{*},z\neq z^{*}\\ \epsilon , & \mathrm{if}\;x\neq x^{*},z=z^{*}\\ (1-\epsilon )/(|Z|-1), & \mathrm{if}\;x\neq x^{*},z\neq z^{*} \end{array}\right.$$

For every variable (T∉{X,Z}) that has parent *Q* on the path P, we assign

$$f^{1}\left(t|q,p_{T}\right)=\left\{\begin{array}{cc} 1-\epsilon , & \mathrm{if}\;q=q^{*},t=t^{*}\\ \epsilon /(|T|-1), & \mathrm{if}\;q=q^{*},t\neq t^{*}\\ \epsilon , & \mathrm{if}\;q\neq q^{*},t=t^{*}\\ (1-\epsilon )/(|T|-1), & \mathrm{if}\;q\neq q^{*},t\neq t^{*} \end{array}\right.$$

We assign the same CPTs for *X* and all variables (T∉{X,Z}) but a different CPT for *Z* in $F^{2}$.

$$f^{2}\left(z|x,p_{Z}\right)=\left\{\begin{array}{cc} \epsilon , & \mathrm{if}\;x=x^{*},z=z^{*}\\ (1-\epsilon )/(|Z|-1), & \mathrm{if}\;x=x^{*},z\neq z^{*}\\ \epsilon , & \mathrm{if}\;x\neq x^{*},z=z^{*}\\ (1-\epsilon )/(|Z|-1), & \mathrm{if}\;x\neq x^{*},z\neq z^{*} \end{array}\right.$$

The two parameterizations ($F^{1}$ and $F^{2}$) induce the same Pr(V), where Pr(v)=0 if $x^{*}\in v$ and Pr(v)>0 otherwise. Next, we show that the parameterization satisfies each positivity constraint (Pr(S|Z)) as long as it does not imply Pr(X)>0. We first show that X∈S implies Pr(X)>0. This is because $\mathrm{Pr}\left(S\right)=\sum_{z}\mathrm{Pr}\left(S|z\right)\mathrm{Pr}\left(z\right)$ and there must exist some instantiation (z) where Pr(z)>0 and Pr(S|z)>0 by constraint. This implies Pr(S)>0 and, therefore, Pr(X)>0. Hence, $C_{V}$ does not contain such a constraint (Pr(S|Z)) where X∈S. Suppose X∈Z; then, Pr(z)>0 if and only if $x^{*}\notin z$. Moreover, since Pr(v)>0, whenever $x^{*}\notin v$, it is guaranteed that Pr(S,z)>0 when Pr(z)>0, which implies Pr(S|Z)>0. Finally, suppose X∉(S∪Z); then, $\mathrm{Pr}\left(S,Z\right)=\sum_{x}\mathrm{Pr}\left(S,Z,x\right)>0$. Hence, the positivity constraint is satisfied by both parameterizations. By construction, ${\mathrm{Pr}}^{1}$ and ${\mathrm{Pr}}^{2}$ induce different values for the causal effect (${\mathrm{Pr}}_{x}\left(y\right)$), since the probability of $Y=y^{*}$ under treatment $do(X=x^{*})$ differs under the two parameterizations. □

**Proposition** **A4.**
*Let G be a causal graph and V be its observed variables. A set of functional variables (W) is consistent with positivity constraints ($C_{V}$) if no single constraint in $C_{V}$ mentions both W∈W and a set ($H_{W}$) that intercepts all directed paths from non-functional variables to W.*

**Proof of Proposition** **A4.** We construct a parameterization (F) and show that the distribution (Pr) induced by F satisfies $C_{V}$, which ensures consistency. The states of each variable (*V*) are represented in the form of $\left(s_{V},p_{1},\cdots ,p_{m}\right)$, where $s_{V}$ and $p_{i}$ (i∈{1,⋯,m}) are both binary indicators (0 or 1). Specifically, each $p_{i}$ corresponds to a “functional descendant path” of *V* defined as follows: A functional descendant path of *V* is a directed path that starts with *V*, and all variables on the path (excluding *V*) are functional. Suppose *V* does not have any functional descendant path; then, the states of *V* are simply represented as $\left(s_{V}\right)$. Next, we show how to assign CPTs for each variable in the causal graph (*G*) based on whether the variable is functional. For each non-functional variable, we assign a uniform distribution. For each functional variable (*W*) whose parents are $T_{1},\cdots ,T_{n}$ and whose functional descendant paths are $P_{1},\cdots ,P_{m}$, we assign the CPT ($f_{W}$) as follows:

(A1) $$\begin{array}{cc} s_{W} & \leftarrow p_{Ind(T_{1},W)}^{T_{1}}⊕\cdots ⊕p_{Ind(T_{n},W)}^{T_{n}}\\ p_{1} & \leftarrow p_{1,1}^{T_{1}}⊕\cdots ⊕p_{n,1}^{T_{n}}\\ \; & \cdots \\ p_{m} & \leftarrow p_{1,m}^{T_{1}}⊕\cdots ⊕p_{n,m}^{T_{n}} \end{array}$$

where $Ind(T_{i},W)$ denotes the index assigned to the path $\left\{\left(T_{i},W\right)\right\}$ (which contains a single edge) in the state of $T^{i}$ and $p_{i,j}^{T_{i}}$ denotes the indicator in the state of $T_{i}$ for functional descendant path $P^{\prime }$ that contains functional descendant path $P_{j}$, i.e., $P^{\prime }=\left\{\left(T_{i},W\right)\right\}\cup P_{j}$. For simplicity, we call the set of variables ($H_{W}$) that satisfies the condition in the proposition a “functional ancestor set” of *W*. We show that Pr(S,Z)>0 for each positivity constraint in the form of Pr(S|Z)>0. Let W⊆S∪Z be a subset of functional variables. Since S∪Z does not contain any functional ancestor set of *W* for each W∈W, it follows that there exist directed paths from a set of non-functional variables ($A_{W}^{\prime }$ to *W*) that are unblocked by M=S∪Z∖{W} and contain only functional variables (excluding $A_{W}^{\prime }$). We can further assume that $A_{W}^{\prime }$ is chosen such that the set $A_{W}=M\cup A_{W}^{\prime }$ forms a valid functional ancestor set for *W*. Next, we show that for any state (*w*) of *W* and instantiation (m) of M, there exists at least one instantiation (a) of $A_{W}^{\prime }$ such that Pr(w,m,a)>0. Let $P^{W}$ denote the set of all directed paths from $A_{W}$ to *W* that do not contain $A_{W}$ (except for the first node on the path). Let $P_{1}^{W}\subseteq P^{W}$ be the paths that start with a variable in M and $P_{2}^{W}\subseteq P^{W}$ be other paths that start with a variable in $A_{W}^{\prime }$. Moreover, for any path (P), let pathval(P) be the binary indicator (e.g., $p_{1}$) for P in the state of P(0) (first variable in P). Since the value assignments for pathval(P) are independent for different Ps, we can always find some instantiation ($a\in A_{W}^{\prime }$) such that the following equality holds given *w* and m:

$$\underset{P_{2}\in P_{2}^{W}}{⨁}\mathrm{pathval}\left(P_{2}\right)=s_{W}⊕\underset{P_{1}\in P_{1}^{W}}{⨁}\mathrm{pathval}\left(P_{1}\right)$$

We next assign values for other path indicators of a such that the indicators for the functional descendant paths in state *w* are set correctly. In particular, for each functional descendant path (P) of *W*, let P be the set of functional descendant paths of $A_{W}$ that do not contain $A_{W}$ (except for the first node on the path) and that contain P as a sub-path. Let $P_{1}\subseteq P$ be the paths that start with a variable in M and $P_{2}\subseteq P$ be other paths that start with a variable in $A_{W}^{\prime }$. Again, since all the indicators for paths in P are independent, we can assign the indicators for $a\in A_{W}^{\prime }$ such that

$$\underset{P_{2}\in P_{2}}{⨁}\mathrm{pathval}\left(P_{2}\right)=\mathrm{pathval}\left(P\right)⊕\underset{P_{1}\in P_{1}}{⨁}\mathrm{pathval}\left(P_{1}\right).$$

Finally, we combine the cases for each individual W∈W by creating the following set: $A_{W}={⋃}_{W\in W}A_{W}$. Since all the functional descendant paths we considered for different *W*s are disjoint, we can always find an assignment (a) for $A_{W}$ that is consistent with the functional dependencies (does not produce any zero probabilities). Consequently, there must exist some full instantiation ((u,v)) compatible with s, z, and a such that Pr(u,v)>0, which implies Pr(s,z)>0. □

**Proof of Proposition** **A1.** Suppose *Y* is not a child of *X* in the CBN; then, $\sum_{X}f_{X}f_{Y}=f_{Y}\left(\sum_{X}f_{X}\right)$, which is guaranteed to be a CPT for *Y*. Suppose *Y* is a child of *X*. Let $P_{X}$ denote the parents of *X* and $P_{Y}$ denote the parents of *Y*, excluding *X*. The new factor ($g=\sum_{X}f_{X}f_{Y}$) is defined over $P_{X}\cup P_{Y}\cup \left\{Y\right\}$. Consider each instantiation ($p_{X}$ and $p_{Y}$); then, $\sum_{y}g\left(p_{X},p_{Y},y\right)=\sum_{y}\sum_{x}f_{X}\left(x|p_{x}\right)f_{Y}\left(y|p_{Y},x\right)=\sum_{x}f_{X}\left(x|p_{X}\right)\sum_{y}f_{Y}\left(y|p_{Y},x\right)=1$. Hence, *g* is a CPT for *Y*. □

**Proof of Proposition** **A2.** Let *X* be the functional variablethat is functionally eliminated. By definition, the elimination only affects the CPTs for the children of *X*. Hence, any functional variable that is not a child of *X* remains functional. For each child (*C*) of *X* that is functional, the new CPT $\left(\sum_{X}f_{X}f_{C}\right)$ only contains values that are either 0 or 1, since both $f_{X}$ and $f_{C}$ are functional. □

**Proof of Proposition** **A3.** First, note that $\pi_{2}$ can always be obtained from $\pi_{1}$ by a sequence of “transpositions”, where each transposition swaps two adjacent variables in the first sequence. Let π be an elimination order, and let $\pi^{\prime }$ be the elimination order resulting from swapping $\pi_{i}=X$ and $\pi_{i+1}=Y$ from the π, i.e.,

$$\pi =(\cdots ,X,Y,\cdots )\;\;\pi^{\prime }=\left(\cdots ,Y,X,\cdots \right)$$

We show that functional elimination according to π and $\pi^{\prime }$ yields the same CBN, which can be applied inductively to conclude that elimination according to $\pi_{1}$ and $\pi_{2}$ yields the same CBN. Since π and $\pi^{\prime }$ agree on the elimination order up to *X*, they yield the same CBN before eliminating variables X,Y. It suffices to show the CBNs resulting from eliminating (X,Y) those resulting from the and elimination of (Y,X) are the same. Let 〈G,Pr〉 be the CBN before eliminating variables X,Y. Suppose *X* and *Y* do not belong to the same family (which contains a variable and its parents), the eliminations of *X* and *Y* are independent, and the order of elimination does not matter. Suppose *X* and *Y* belong to the same family; then, they are either parent and child or co-parents (*X* and *Y* are co-parents if they have a same child). Without loss of generality, suppose *X* is a parent of *Y*. Eliminating (X,Y) and eliminating (Y,X) yield the same causal graph that is defined as follows. Each child (*C*) of *Y* has parents $P_{X}\cup P_{Y}\cup P_{C}\setminus \left\{X,Y\right\}$, and any other child *C* of *X* has parents $P_{X}\cup P_{C}\setminus \left\{X\right\}$. We next consider the CPTs. For each common child (*C*) of *X* and *Y*, its CPT resulting from eliminating (X,Y) is $f_{C}^{1}=\sum_{Y}\left(\sum_{X}f_{C}f_{X}\right)\left(\sum_{X}f_{Y}f_{X}\right)$, and the CPT resulting from eliminating (Y,X) is $f_{C}^{2}=\sum_{X}f_{X}\left(\sum_{Y}f_{C}f_{Y}\right)$. Since *X* is a parent of *Y*, we have $Y\notin f_{X}$ and

$$\begin{array}{cc} f_{C}^{2} & =\sum_{X}\sum_{Y}f_{X}f_{C}f_{Y}=\sum_{Y}\sum_{X}f_{X}f_{C}f_{Y}\\ \; & =\sum_{Y}\left(\sum_{X}f_{X}f_{C}\right)\left(\sum_{X}f_{X}f_{Y}\right)=f_{C}^{1} \end{array}$$

Next, we consider the case when *C* is a child of *Y* but not a child of *X*. The CPT for *C* resulting from eliminating (X,Y) is $f_{C}^{1}=\sum_{Y}f_{C}\left(\sum_{X}f_{Y}f_{X}\right)$, and the CPT resulting from eliminating (Y,X) is $f_{C}^{2}=\sum_{X}f_{X}\left(\sum_{Y}f_{C}f_{Y}\right)$. Again, since *X* is a parent of *Y*, we have $Y\notin f_{X}$ and

$$\begin{array}{cc} f_{C}^{2} & =\sum_{X}\sum_{Y}f_{X}f_{C}f_{Y}=\sum_{Y}\sum_{X}f_{X}f_{C}f_{Y}\\ \; & =\sum_{Y}f_{C}\left(\sum_{X}f_{X}f_{Y}\right)=f_{C}^{1} \end{array}$$

Finally, we consider the case when *C* is a child of *X* but not a child of *Y*. Regardless of the order of *X* and *Y*, the CPT for *C* resulting from eliminating *X* and *Y* is $\left(\sum_{X}f_{X}f_{C}\right)$. Next, we consider the case when *X* and *Y* are co-parents. Regardless of the order of *X* and *Y*, the causal graph resulting from the elimination satisfies the following properties: (1) for each common child (*C*) of *X* and *Y*, the parents for *C* are $P_{X}\cup P_{Y}\cup P_{C}\setminus \left\{X,Y\right\}$; (2) the parents of each *C* that is a child of *X* but not a child of *Y* are $P_{X}\cup P_{C}\setminus \left\{X\right\}$; and (3) the parents of each *C* that is a child of *Y* but not a child of *X* are $P_{Y}\cup P_{C}\setminus \left\{Y\right\}$. Next, we consider the CPTs. The CPT for each common child *C* of *X* and *Y* resulting from eliminating (X,Y) is $f_{C}^{1}=\sum_{Y}f_{Y}\left(\sum_{X}f_{X}f_{C}\right)$, and the CPT resulting from eliminating (Y,X) is $f_{C}^{2}=\sum_{X}f_{X}\left(\sum_{Y}f_{Y}f_{C}\right)$. Since *X* and *Y* are not parent and child, we have $X\notin f_{Y}$, $Y\notin f_{X}$, and

$$f_{C}^{1}=\sum_{Y}\sum_{X}f_{Y}f_{X}f_{C}=\sum_{X}\sum_{Y}f_{Y}f_{X}f_{C}=f_{C}^{2}$$

For each *C* that is a child of *X* but not a child of *Y*, regardless of the order of *X* and *Y*, the CPT for *C* resulting from eliminating variables *X* and *Y* is $\left(\sum_{X}f_{X}f_{C}\right)$. A similar result holds for each *C* that is a child of *Y* but not a child of *X*. □

**Proof of Theorem** **A1.** It suffices to show that ${\mathrm{Pr}}^{\prime }=\sum_{X}\mathrm{Pr}$ when we eliminate a single variable (*X*). Let F denote the set of CPTs for M. Since $f_{X}$ is a functional CPT for *X*, we can replicate $f_{X}$ in F, which yields a new CPT set (replication) ($F^{\prime }$) that induces the same distribution as F (see details in ([21], Theorem 4)). Specifically, we pair the CPT for each child (*C*) of *X* with an extra copy of $f_{X}$, denoted as $\left(f_{X},f_{C}\right)$, which yields a list of pairs ($\left(f_{X},f_{C_{1}}\right),\cdots ,\left(f_{X},f_{C_{k}}\right)$, where $C_{1},\cdots ,C_{k}$ are the children of *X*). Functionally eliminating *X* from $F^{\prime }$ yields ([21], Corollary 1)

(A2) $$\begin{array}{cc} \sum_{X}\mathrm{Pr}=\sum_{X}F^{\prime } & =H\cdot \left(\sum_{X}f_{X}f_{C_{1}}\right)\cdots \left(\sum_{X}f_{X}f_{C_{k}}\right)\\ \; & =H\cdot f_{C_{1}}^{\prime }\cdots f_{C_{k}}^{\prime }={\mathrm{Pr}}^{\prime } \end{array}$$

where H represent the CPTs in F that do not contain *X* and each $f_{C_{i}}^{\prime }$ is the CPT for child $C_{i}$ in $M^{\prime }$. □

*Proof of Theorem A2*

**Lemma** **A1.**
*Consider a CBN 〈G,F〉 and its mutilated CBN $\langle G_{x},F_{x}\rangle$ under do(x). Let W be a functional variable not in X and let $\langle G^{\prime },F^{\prime }\rangle$ and $\langle G_{x}^{\prime },F_{x}^{\prime }\rangle$ be the results of functionally eliminating W from 〈G,F〉 and $\langle G_{x},F_{x}\rangle$, respectively. Then, $\langle G_{x}^{\prime },F_{x}^{\prime }\rangle$ is the mutilated CBN for $\langle G^{\prime },F^{\prime }\rangle$.*

**Proof.** First, observe that the children of *W* in *G* and $G_{x}$ can only differ by the variables in X. Let $C_{1}$ be the children of *W* in both *G* and $G_{x}$, and let $C_{2}$ be the children of *W* in *G* but not in $G_{x}$. According to the definition of mutilated CBN, *W* has the same set of parents and CPT in 〈G,F〉 and $\langle G_{x},F_{x}\rangle$. Similarly, each child ($C\in C_{1}$) has the same set of parents and CPT in 〈G,F〉 and $\langle G_{x},F_{x}\rangle$. Hence, eliminating *W* yields the same set of parents and CPT for each C∈C in $\langle G^{\prime },F^{\prime }\rangle$ and $\langle G_{x}^{\prime },F_{x}^{\prime }\rangle$. Next, we consider the set of parents and CPT for each child ($C\in C_{2}$). Since *W* is not a parent of *C* in $\langle G_{x},F_{x}\rangle$, variable *C* has the same set of parents and CPT in $\langle G_{x}^{\prime },F_{x}^{\prime }\rangle$. Exactly same set of parents (empty) and CPT are assigned to *C* in the mutilated CBN for $\langle G^{\prime },F^{\prime }\rangle$. □

**Proof of Theorem** **A2.** Consider a CBN 〈G,F〉 and its mutilated CBN $\langle G_{x},F_{x}\rangle$. Let Pr and ${\mathrm{Pr}}_{x}$ be the distributions induced by F and $F_{x}$ over variables V, respectively. According to Lemma A1, we can eliminate each W∈W inductively from 〈G,F〉 and $\langle G_{x},F_{x}\rangle$ and obtain $\langle G^{\prime },F^{\prime }\rangle$ and its mutilated CBN $\langle G_{x}^{\prime },F_{x}^{\prime }\rangle$. According to Theorem A1, the distribution induced by $F_{x}^{\prime }$ is exactly $\sum_{W}{\mathrm{Pr}}_{x}\left(V\right)$. □

**Proof of Proposition** **2.** First, note that the extended set ($Z^{\prime }$) contains Z and all variables that are functionally determined by Z. Consider any path (P) between some X∈X and Y∈Y. We show that P is blocked by $Z^{\prime }$ iff it is blocked by Z according to the definition presented in [40]. We first show the if part. Suppose there is a convergent valve (see [38] (Ch. 4) for more details on convergent, divergent, and sequential valves) for a variable *W* that is closed when conditioned on Z; then, the valve is still closed when conditioned on $Z^{\prime }$ unless the parents of *W* are in $Z^{\prime }$. However, the path (P) is blocked in the latter case, since the parents of *W* must have sequential/divergent valves. Suppose there is a sequential/divergent valve that is closed when conditioned on Z according to [40]; then, *W* must be in $Z^{\prime }$, since it is functionally determined by Z. Hence, the valve is also closed when conditioned on $Z^{\prime }$. Next, we show the only–if part. Suppose a convergent valve for variable *W* is closed when conditioned on $Z^{\prime }$; then, none of Z is a descendant of *W*, since $Z^{\prime }$ is a superset of Z. Suppose a sequential/divergent valve for variable *W* is closed when conditioned on $Z^{\prime }$; then, *W* is functionally determined by Z by the construction of $Z^{\prime }$. Thus, the valve is closed as described in [40]. □

**Proof of Theorem** **1.** By induction, it suffices to show that X and Y are D-separated by Z in 〈G,W〉 iff they are D-separated by Z in $\langle G^{\prime },W^{\prime }\rangle$, where $G^{\prime }$ is the result of functionally eliminating a single variable (T∈W) from $G^{\prime }$ and $W^{\prime }=W\setminus \left\{T\right\}$. We first show the contrapositive of the if part. Suppose X and Y are not D-separated by Z in 〈G,W〉; owing to the completeness of D-separation, there exists a parameterization (F) on *G* such that ${\left(X\;/\;⊥\;\;\;⊥Y|Z\right)}_{F}$. If we eliminate *T* from the CBN 〈G,F〉, we obtain another CBN $\langle G^{\prime },F^{\prime }\rangle$, where $F^{\prime }$ is the parameterization for $G^{\prime }$. According to Theorem A1, the marginal probabilities are preserved for the variables in $G^{\prime }$, which include X,Y,Z. Hence, ${\left(X\;/\;⊥\;\;\;⊥Y|Z\right)}_{F^{\prime }}$ and X and Y are not D-separated by Z in $\langle G^{\prime },W^{\prime }\rangle$. Next consider the contrapositive of the only–if part. Suppose X and Y are not D-separated by Z in $\langle G^{\prime },W^{\prime }\rangle$; then, there exists a parameterization ($F^{\prime }$) of $G^{\prime }$ such that ${\left(X\;/\;⊥\;\;\;⊥Y|Z\right)}_{F^{\prime }}$ owing to the completeness of D-separation. We construct a parameterization (F) for *G* such that $F^{\prime }$ is the parameterization of $G^{\prime }$, which results from eliminating *T* from the CBN 〈G,F〉. This is sufficient to show that X and Y are not D-separated by Z in 〈G,W〉, since the marginals are preserved by Theorem A1.

**Construction Method** Let $P_{T}$ and $C_{T}$ denote the parents and children of *T* in *G*, respectively. Our construction assumes that the cardinality of *T* is the number of instantiations for its parents ($P_{T}$), that is, there is a one-to-one correspondence between the states of *T* and the instantiations of $P_{T}$, and we use α(t) to denote the instantiation ($p_{T}$) corresponding to state *t*. The functional CPT for *T* is assigned as $f_{T}\left(t|p_{T}\right)=1$ if $\alpha \left(t\right)=p_{T}$ and $f_{T}\left(t|p_{T}\right)=0$ otherwise for each instantiation ($p_{T}$) of $P_{T}$. Now, consider each child ($C\in C_{T}$) that has parents $P_{C}$ (excluding *T*) and *T* in *G*. It immediately follows from Definition 8 that *C* has parents $P_{T}\cup P_{C}$ in $G^{\prime }$. Next, we construct the CPT ($f_{C}$) in F based on its CPT ($f_{C}^{\prime }$) in $F^{\prime }$. Consider each parent instantiation $\left(t,p_{C}\right)$, where *t* is a state of *T* and $p_{C}$ is an instantiation of $P_{C}$. If α(t) is consistent with $p_{C}$, $f_{C}\left(c|t,p_{C}\right)=f_{C}^{\prime }\left(c|\alpha \left(t\right),p_{C}\right)$ is assigned for each state (*c*) (For clarity, we use the notation | to separate a variable and its parents in a CPT). Otherwise, any functional distribution is assigned for $f_{C}\left(C|t,p_{C}\right)$. This construction ensures that the constructed CPT ($f_{T}$) for *T* is functional and that the functional dependencies among other variables are preserved. In particular, for each child (*C*) of *T*, the constructed CPT ($f_{C}$) is functional iff $f_{C}^{\prime }$ is functional. This construction method is reused later in other proofs.

Now, we just need to show that CBN $\langle G^{\prime },F^{\prime }\rangle$ is the result of eliminating *T* from the (constructed) CBN 〈G,F〉. In particular, we need to check that the CPT for each child ($C\in C_{T}$) in $F^{\prime }$ is correctly computed from the constructed CPTs in F. For each instantiation $\left(p_{T},p_{C}\right)$ and state (*c*) of *C*,

$$\begin{array}{cc} f_{C}^{\prime }\left(c|p_{T},p_{C}\right) & =f_{C}\left(c|t^{*},p_{C}\right)=f_{T}\left(t^{*}|p_{T}\right)f_{C}\left(c|t^{*},p_{C}\right)\\ \; & =\sum_{t}f_{T}\left(t|p_{T}\right)f_{C}\left(c|t,p_{C}\right) \end{array}$$

where $t^{*}$ is the state of *T* such that $\alpha \left(t^{*}\right)=p_{T}$. □

**Proof of Theorem** **2.** We prove the theorem by induction. It suffices to show the following statement: For each causal graph (*G*) with observed variables (V) and functional variables (W), the causal effect (${\mathrm{Pr}}_{x}\left(Y\right)$) is F-identifiable with respect to $\langle G,V,C_{V},W\rangle$ iff it is F-identifiable with respect to $\langle G^{\prime },V,C_{V},W^{\prime }\rangle$, where $G^{\prime }$ is the result of functionally eliminating some hidden functional variable (T∈W) and $W^{\prime }=W\setminus \left\{T\right\}$. We first show the contrapositive of the if part. Suppose ${\mathrm{Pr}}_{x}\left(Y\right)$ is not F-identifiable with respect to $\langle G,V,C_{V},W\rangle$; then, there exist two CBNs ($\langle G,F_{1}\rangle$ and $\langle G,F_{2}\rangle$) that induce distributions (${\mathrm{Pr}}_{1},{\mathrm{Pr}}_{2}$) such that ${\mathrm{Pr}}_{1}\left(V\right)={\mathrm{Pr}}_{2}\left(V\right)$ but ${\mathrm{Pr}}_{1x}\left(Y\right)\neq {\mathrm{Pr}}_{2x}\left(Y\right)$. Let $\langle G^{\prime },F_{1}^{\prime }\rangle$ and $\langle G^{\prime },F_{2}^{\prime }\rangle$ be the results of eliminating T∉V from $\langle G,F_{1}\rangle$ and $\langle G,F_{2}\rangle$, respectively; the two CBNs attain the same marginal distribution on V but different causal effects according to Theorems A1 and A2. Hence, ${\mathrm{Pr}}_{x}\left(Y\right)$ is not F-identifiable with respect to $\langle G^{\prime },V,C_{V},W^{\prime }\rangle$ either. Next, we show the contrapositive of the only–if part. Suppose ${\mathrm{Pr}}_{x}\left(Y\right)$ is not F-identifiable with respect to $\langle G^{\prime },V,C_{V},W^{\prime }\rangle$; then, there exist two CBNs ($\langle G^{\prime },F_{1}^{\prime }\rangle$ and $\langle G^{\prime },F_{2}^{\prime }\rangle$) that induce distributions (${\mathrm{Pr}}_{1}^{\prime },{\mathrm{Pr}}_{2}^{\prime }$) such that ${\mathrm{Pr}}_{1}^{\prime }\left(V\right)={\mathrm{Pr}}_{2}^{\prime }\left(V\right)$ but ${\mathrm{Pr}}_{1x}^{\prime }\left(Y\right)\neq {\mathrm{Pr}}_{2x}^{\prime }\left(Y\right)$. We can obtain $\langle G,F_{1}\rangle$ and $\langle G,F_{2}\rangle$ by, agains, considering the construction method outlined in Theorem 1, where we assign a one-to-one mapping for *T* and adopt the CPTs from $F_{1}^{\prime }$ and $F_{2}^{\prime }$ for the children of *T*. This way, $\langle G^{\prime },F_{1}^{\prime }\rangle$ and $\langle G^{\prime },F_{2}^{\prime }\rangle$ become the results of eliminating *T* from the constructed $\langle G,F_{1}\rangle$ and $\langle G,F_{2}\rangle$, respectively. Since T∉V, ${\mathrm{Pr}}_{1}\left(V\right)={\mathrm{Pr}}_{1}^{\prime }\left(V\right)={\mathrm{Pr}}_{2}^{\prime }\left(V\right)={\mathrm{Pr}}_{2}\left(V\right)$ according to Theorem A1 and ${\mathrm{Pr}}_{1x}\left(Y\right)={\mathrm{Pr}}_{1x}^{\prime }\left(Y\right)\neq {\mathrm{Pr}}_{2x}^{\prime }\left(Y\right)={\mathrm{Pr}}_{2x}\left(Y\right)$ according to Theorem A2. Hence, ${\mathrm{Pr}}_{x}\left(Y\right)$ is not F-identifiable with respect to $\langle G,V,C_{V},W\rangle$ either. □

**Proof of Theorem** **3.** Since we only functionally eliminate variables that have observed parents, it is guaranteed that each Z∈Z has observed parents when it is eliminated. By induction, it suffices to show that ${\mathrm{Pr}}_{x}\left(Y\right)$ is F-identifiable with respect to $\langle G,V,C_{V},W\rangle$ iff it is F-identifiable with respect to $\langle G^{\prime },V^{\prime },C_{V},W^{\prime })$, where $G^{\prime }$ is the result of eliminating a single functional variable (Z∈W) with observed parents from *G*, $V^{\prime }=V\setminus \left\{Z\right\}$, and $W^{\prime }=W\setminus \left\{Z\right\}$. We first show the contrapositive of the if part. Suppose ${\mathrm{Pr}}_{x}\left(Y\right)$ is not F-identifiable with respect to $\langle G,V,C_{V},W\rangle$; then, there exist two CBNs ($\langle G,F_{1}\rangle$ and $\langle G,F_{2}\rangle$) that induce distributions ${\mathrm{Pr}}_{1},{\mathrm{Pr}}_{2}$, where ${\mathrm{Pr}}_{1}\left(V\right)={\mathrm{Pr}}_{2}\left(V\right)$ but ${\mathrm{Pr}}_{1x}\left(Y\right)\neq {\mathrm{Pr}}_{2x}\left(Y\right)$. Let $\langle G^{\prime },F_{1}^{\prime }\rangle$ and $\langle G^{\prime },F_{2}^{\prime }\rangle$ be the results of eliminating *Z* from $\langle G,F_{1}\rangle$ and $\langle G,F_{2}\rangle$, respectively; the two CBNs induce the same marginal distribution (${\mathrm{Pr}}_{1}^{\prime }\left(V^{\prime }\right)={\mathrm{Pr}}_{2}^{\prime }\left(V^{\prime }\right)$) according to Theorem A1 but different causal effects (${\mathrm{Pr}}_{1x}^{\prime }\left(Y\right)\neq {\mathrm{Pr}}_{2x}^{\prime }\left(Y\right)$) according to Theorem A2. Hence, ${\mathrm{Pr}}_{x}\left(Y\right)$ is not F-identifiable with respect to $\langle G^{\prime },V^{\prime },C_{V},W^{\prime }\rangle$. We now consider the contrapositive of the only–if part. Suppose ${\mathrm{Pr}}_{x}\left(Y\right)$ is not F-identifiable with respect to $\langle G^{\prime },V^{\prime },C_{V},W^{\prime }\rangle$; then, there exist two CBNs ($\langle G^{\prime },F_{1}^{\prime }\rangle$ and $\langle G^{\prime },F_{2}^{\prime }\rangle$) that induce distributions (${\mathrm{Pr}}_{1}^{\prime },{\mathrm{Pr}}_{2}^{\prime }$) such that ${\mathrm{Pr}}_{1}^{\prime }\left(V^{\prime }\right)={\mathrm{Pr}}_{2}^{\prime }\left(V^{\prime }\right)$ but ${\mathrm{Pr}}_{1x}^{\prime }\left(Y\right)\neq {\mathrm{Pr}}_{2x}^{\prime }\left(Y\right)$. We, again, consider the construction method from the proof of Theorem 1, which produces two CBNs ($\langle G,F_{1}\rangle$ and $\langle G,F_{2}\rangle$). Moreover, $\langle G^{\prime },F_{1}^{\prime }\rangle$ and $\langle G^{\prime },F_{2}^{\prime }\rangle$ are the results of eliminating *Z* from $\langle G,F_{1}\rangle$ and $\langle G,F_{2}\rangle$, respectively. It is guaranteed that the two constructed CBNs produce different causal effects (${\mathrm{Pr}}_{1x}\left(Y\right)={\mathrm{Pr}}_{1x}^{\prime }\left(Y\right)\neq {\mathrm{Pr}}_{2x}^{\prime }\left(Y\right)={\mathrm{Pr}}_{2x}\left(Y\right)$) according to Theorem A2. We need to show that $\langle G,F_{1}\rangle$ and $\langle G,F_{2}\rangle$ induce the same distribution over variables of $V=V^{\prime }\cup \left\{Z\right\}$. Consider any instantiation $\left(v^{\prime },z\right)$ of V. Since $F_{1}$ and $F_{2}$ assign the same one-to-one mapping (α) between *Z* and its parents in *G*, it is guaranteed that the probabilities are ${\mathrm{Pr}}_{1}\left(v^{\prime },z\right)={\mathrm{Pr}}_{2}\left(v^{\prime },z\right)=0$, except for the single state ($z^{*}$) where $\alpha (z^{*})=p$, where p is the parent instantiation of *Z*, consistent with $v^{\prime }$. By construction, ${\mathrm{Pr}}_{1}\left(v^{\prime },z^{*},u\right)={\mathrm{Pr}}_{1}^{\prime }\left(v^{\prime },u\right)$ for every instantiation (u) of hidden variables (U). Similarly, ${\mathrm{Pr}}_{2}\left(v^{\prime },z^{*},u\right)={\mathrm{Pr}}_{2}^{\prime }\left(v^{\prime },u\right)$ for every instantiation $\left(v^{\prime },z,u\right)$. It then follows that ${\mathrm{Pr}}_{1}\left(v^{\prime },z^{*}\right)=\sum_{u}{\mathrm{Pr}}_{1}\left(v^{\prime },z^{*},u\right)=\sum_{u}{\mathrm{Pr}}_{1}^{\prime }\left(v^{\prime },u\right)={\mathrm{Pr}}_{1}^{\prime }\left(v^{\prime }\right)={\mathrm{Pr}}_{2}^{\prime }\left(v^{\prime }\right)=\sum_{u}{\mathrm{Pr}}_{2}^{\prime }\left(v^{\prime },u\right)=\sum_{u}{\mathrm{Pr}}_{2}\left(v^{\prime },z^{*},u\right)={\mathrm{Pr}}_{2}\left(v^{\prime },z^{*}\right)$. This means ${\mathrm{Pr}}_{x}\left(Y\right)$ is not F-identifiable with respect to $\langle G,V,C_{V},W\rangle$ either. □

*Proof of Theorem 4*

**Lemma** **A2.**
*Let G be a causal graph, V be its observed variables, and W be its functional variables. Let Z be a non-descendant of W that has at least one hidden parent; then, a causal effect is F-identifiable with respect to $\langle G,V,C_{V},W\rangle$ iff it is F-identifiable with respect to $\langle G,V,C_{V},W\cup \left\{Z\right\}\rangle$.*

**Proof.** Let $W^{\prime }$ denote the set expressed as W∪{Z}. The only–if part holds immediately due to the fact that every distribution that can possibly be induced from $\langle G,W^{\prime }\rangle$ can also be induced from 〈G,W〉. Next, we consider the contrapositive of the if part. Suppose a causal effect is not F-identifiable with respect to $\langle G,V,C_{V},W\rangle$; then, there exist two CBNs ($\langle G,F_{1}\rangle$ and $\langle G,F_{2}\rangle$) that induce distributions (${\mathrm{Pr}}_{1},{\mathrm{Pr}}_{2}$) such that ${\mathrm{Pr}}_{1}\left(V\right)={\mathrm{Pr}}_{2}\left(V\right)$ but ${\mathrm{Pr}}_{1x}\left(Y\right)\neq {\mathrm{Pr}}_{2x}\left(Y\right)$. Next, we construct $\langle G,F_{1}^{\prime \prime }\rangle$ and $\langle G,F_{2}^{\prime \prime }\rangle$, which constitute an example of unidentifiability with respect to $\langle G,V,C_{V},W^{\prime }\rangle$. In particular, the CPTs for *Z* need to be functional in the constructed CBNs. Without losing generality, we show the construction of $\langle G,F_{1}^{\prime \prime }\rangle$ from $\langle G,F_{1}\rangle$, which involves two steps (the construction of $\langle G,F_{2}^{\prime \prime }\rangle$ from $\langle G,F_{2}\rangle$ follows the same procedure). Let P be the parents of *Z*. The first step constructs a CBN $\langle G^{\prime },F_{1}^{\prime }\rangle$ based on the known method that transforms any (non-functional) CPT into a functional CPT. This is done by adding an auxiliary hidden root parent (*U*) for *Z* whose states correspond to the possible functions between P and *Z*. The CPTs for *U* and *Z* are assigned accordingly such that $f_{Z}=\sum_{U}f_{U}^{\prime }f_{Z}^{\prime }$, where $f_{U}^{\prime }$ and $f_{Z}^{\prime }$ are the constructed CPTs in $F_{1}^{\prime }$ (Each state (*u*) of *U* corresponds to a function ($\gamma_{u}$), where $\gamma_{u}\left(p\right)$ is mapped to some state of *Z* for each instantiation (p). Thus, variable *U* has ${\left|Z\right|}^{\left|P\right|}$ states, since there is a total of ${\left|Z\right|}^{\left|P\right|}$ possible functions from P to *Z*. For each instantiation (z,u,p), the functional CPT for *Z* is defined as $f_{Z}^{\prime }\left(z|u,p\right)=1$ if $z=\gamma_{u}\left(p\right)$ and $f_{Z}^{\prime }\left(z|u,p\right)=0$ otherwise. The CPT for *U* is assigned as $f_{U}^{\prime }\left(u\right)=\prod_{p\in P}f_{Z}\left(\gamma_{u}\left(p\right)|p\right)$). It follows that $F_{1}$ and $F_{1}^{\prime }$ induce the same distribution over V, since $F_{1}=\sum_{U}F_{1}^{\prime }$. The causal effect is also preserved, since the summing out of *U* is independent of other CPTs in the mutilated CBN for $\langle G^{\prime },F_{1}^{\prime }\rangle$. Our second step involves converting the CBN $\langle G^{\prime },F_{1}^{\prime }\rangle$ (constructed in the first step) into the CBN $\langle G,F_{1}^{\prime \prime }\rangle$ over the original graph (*G*). Let T∈P be the hidden parent of *W* in *G*. We merge the auxiliary parents (*U* and *T*) into a new variable ($T^{\prime }$) and substitute it for *T* in *G*, i.e., $T^{\prime }$ has the same parents and children as *T*. $T^{\prime }$ is constructed as the Cartesian product of *U* and *T*. Each state of $T^{\prime }$ is represented as a pair (u,t), where *u* is a state of *U* and *t* is a state of *T*. We are ready to assign new CPTs for $T^{\prime }$ and its children. For each parent instantiation (p) of P and each state ((u,t)) of $T^{\prime }$, we assign the CPT for $T^{\prime }$ in $F^{\prime \prime }$ as $f_{T^{\prime }}^{\prime \prime }\left(\left(u,t\right)|p\right)=f_{U}^{\prime }\left(u\right)f_{T}^{\prime }\left(t|p\right)$. Next, consider each child (*C*) of $T^{\prime }$ that has parents ($P_{C}$) (excluding $T^{\prime }$). For each instantiation ($p_{C}$) of $P_{C}$ and each state (u,t) of $T^{\prime }$, we assign the CPT for *C* in $F^{\prime \prime }$ as $f_{C}^{\prime \prime }\left(c|p_{C},\left(u,t\right)\right)=f_{C}^{\prime }\left(c|p_{C}\right)$ for each state (*c*). Note that $f_{C}^{\prime \prime }$ is functional iff $f_{C}^{\prime }$ is functional. Hence, the CPTs for W are all functional in $F^{\prime \prime }$. We need to show that $\langle G,F_{1}^{\prime \prime }\rangle$ preserves the distribution on V and the causal effect from $\langle G^{\prime },F_{1}^{\prime }\rangle$. Let $U^{\prime \prime }$ be the hidden variables in $\langle G,F_{1}^{\prime \prime }\rangle$ and $U^{\prime }$ be the hidden variables in $\langle G^{\prime },F_{1}^{\prime }\rangle$. The distribution on V is preserved, since there is a one-to-one correspondence between each instantiation $\left(v,u^{\prime \prime }\right)$ in $\langle G,F_{1}^{\prime \prime }\rangle$ and each instantiation $\left(v,u^{\prime }\right)$ in $\langle G^{\prime },F_{1}^{\prime }\rangle$, where the two instantiations agree on v and are assigned with the same probability, i.e., ${\mathrm{Pr}}_{1}^{\prime \prime }\left(v,u^{\prime \prime }\right)={\mathrm{Pr}}_{1}^{\prime }\left(v,u^{\prime }\right)$. Hence, ${\mathrm{Pr}}_{1}^{\prime \prime }\left(v\right)=\sum_{u^{\prime \prime }}{\mathrm{Pr}}_{1}^{\prime \prime }\left(v,u^{\prime \prime }\right)=\sum_{u^{\prime }}{\mathrm{Pr}}_{1}^{\prime }\left(v,u^{\prime }\right)={\mathrm{Pr}}_{1}^{\prime }\left(v\right)$ for every instantiation (v). The preservation of the causal effect can be shown similarly but on the mutilated CBNs. Thus, $\langle G,F_{1}^{\prime \prime }\rangle$ preserves both the distribution on V and the causal effect from $\langle G,F_{1}\rangle$. Similarly, we can construct $\langle G,F_{2}^{\prime \prime }\rangle$, which preserves the distribution on V and the causal effect from $\langle G,F_{2}\rangle$. The two CBNs ($\langle G,F_{1}^{\prime \prime }\rangle$ and $\langle G,F_{2}^{\prime \prime }\rangle$) constitute an example of unidentifiability with respect to $\langle G,V,C_{V},W^{\prime }\rangle$. □

**Proof of Theorem** **4.** We prove the theorem by induction. We first order all the functional variables in a bottom-up order. Let $W^{i}$ denote the *i*th functional variable in the order and $W^{\left(i\right)}$ denote the functional variables that are ordered before $W^{i}$ (including $W^{i}$). It follows that we can go over each $W^{i}$ in the order and show that a causal effect is F-identifiable with respect to $\langle G,V,C_{V},W^{\left(i-1\right)}\rangle$ iff it is F-identifiable with respect to $\langle G,V,C_{V},W^{\left(i\right)}\rangle$ according to Lemma A2. Since F-identifiability with respect to $\langle G,V,C_{V},W^{\left(0\right)}\rangle$ is equivalent to identifiability with respect to $\langle G,V,C_{V}\rangle$, we conclude that the causal effect is F-identifiable with respect to $\langle G,V,C_{V},W\rangle$ iff it is identifiable with respect to $\langle G,V,C_{V}\rangle$. □

*Proof of Theorem 5*

The proof of the theorem is organized as follows. We start with a lemma (Lemma A3) that allows us to modify the CPT of a variable when the marginal probability over its parents contains zero entries. We then show a main lemma (Lemma A4) that allows us to reduce F-identifiability to identifiability when all functional variables are observed or have a hidden parent. Finally, we prove the theorem based on the main lemma and previous theorems.

**Lemma** **A3.**
*Consider two CBNs that have the same causal graph and induce distributions ${\mathrm{Pr}}_{1}$ and ${\mathrm{Pr}}_{2}$. Suppose the CPTs of the two CBNs only differ by $f_{X}\left(X,P\right)$. Then, ${\mathrm{Pr}}_{1}\left(p\right)=0$ iff ${\mathrm{Pr}}_{2}\left(p\right)=0$ for all instantiations (p) of P.*

**Proof.** Let $f_{Y}$ and $g_{Y}$ denote the CPTs for *Y* in the first and second CBN, respectively. Let An(P) be the ancestors of variables in P (including P). If we eliminate all variables other than An(P), then we obtain the factor set expressed as $F_{1}=\prod_{Y\in An(P)}f_{Y}$ for the first CBN and that expressed as $F_{2}=\prod_{Y\in An(P)}g_{Y}$ for the second CBN. Since all CPTs are the same for variables in An(P), it is guaranteed that $F_{1}=F_{2}$. If we further eliminate variables other than P from $F_{1}$ and $F_{2}$, we obtain marginal distributions of ${\mathrm{Pr}}_{1}\left(P\right)={\sum^{=}}_{P}F_{1}$ and ${\mathrm{Pr}}_{2}\left(P\right)={\sum^{=}}_{P}F_{2}$, where ${\sum^{=}}_{P}$ denotes the projection operation that sums out variables other than P from a factor. Hence, ${\mathrm{Pr}}_{1}\left(P\right)={\mathrm{Pr}}_{2}\left(P\right)$, which concludes the proof. □

**Lemma** **A4.**
*If a causal effect is F-identifiable with respect to $\langle G,V,C_{V},W\rangle$ but is not identifiable with respect to $\langle G,V,C_{V}\rangle$, then there must exist at least one functional variable that is hidden and whose parents are all observed.*

**Proof.** The lemma is the same as saying that if every functional variable is observed or has a hidden parent, then F-identifiability is equivalent to identifiability. We go over each functional variable ($W_{i}\in W$) in a bottom-up order (Π) and prove the following inductive statement: A causal effect (${\mathrm{Pr}}_{x}\left(Y\right)$) is F-identifiable with respect to $\langle G,V,C_{V},W^{\left(i\right)}\rangle$ iff it is F-identifiable with respect to $\langle G,V,C_{V},W^{\left(i-1\right)}\rangle$, where $W^{\left(i\right)}$ is a subset of variables in W that are ordered before $W_{i}$ (and including $W_{i}$) in Π. Note that $W^{\left(0\right)}=\emptyset$ and F-identifiability with respect to $\langle G,V,C_{V},W^{\left(0\right)}\rangle$ collapses into identifiability with respect to $\langle G,V,C_{V}\rangle$. The if part follows from the definitions of identifiability and F-identifiability. Next, we consider the contrapositive of the only–if part. Let *Z* be the functional variable in W that is considered in the current inductive step. Let $\langle G,F_{1}\rangle$ and $\langle G,F_{2}\rangle$ be the two CBNs inducing distributions ${\mathrm{Pr}}_{1}$ and ${\mathrm{Pr}}_{2}$, which constitute the unidentifiability, i.e., ${\mathrm{Pr}}_{1}\left(V\right)={\mathrm{Pr}}_{2}\left(V\right)$ and ${\mathrm{Pr}}_{1x}\left(Y\right)\neq {\mathrm{Pr}}_{2x}\left(Y\right)$. Our goal is to construct two CBNs ($\langle G,F_{1}^{\prime \prime \prime }\rangle$ and $\langle G,F_{2}^{\prime \prime \prime }\rangle$) that induce distributions ${\mathrm{Pr}}_{1}^{\prime \prime \prime }$ and ${\mathrm{Pr}}_{2}^{\prime \prime \prime }$ and contain functional CPTs for *Z* such that ${\mathrm{Pr}}_{1}^{\prime \prime \prime }\left(V\right)={\mathrm{Pr}}_{2}^{\prime \prime \prime }\left(V\right)$ and ${\mathrm{Pr}}_{1x}^{\prime \prime \prime }\left(Y\right)\neq {\mathrm{Pr}}_{2x}^{\prime \prime \prime }\left(Y\right)$. Suppose *Z* has a hidden parent; we directly employ Lemma A2 to construct the two CBNs. Next, we consider the case when *Z* is observed and has observed parents. By default, we use $f_{Z}$ to $g_{Z}$ to denote the CPTs for *Z* in $F_{1}$ and $F_{2}$. The following three steps are considered to construct an instance of unidentifiability.

**First Step:** We construct $\langle G,F_{1}^{\prime }\rangle$ and $\langle G,F_{2}^{\prime }\rangle$ by modifying the CPTs for *Z*. Let P be the parents of *Z* in *G*. For each instantiation (p) of P where ${\mathrm{Pr}}_{1}\left(p\right)={\mathrm{Pr}}_{2}\left(p\right)=0$, we modify entries $f_{Z}^{\prime }\left(Z|p\right)$ and $g_{Z}^{\prime }\left(Z|p\right)$ for CPTs $f_{Z}^{\prime }$ and $g_{Z}^{\prime }$ in $F_{1}^{\prime }$ and $F_{2}^{\prime }$ as follows. Since ${\mathrm{Pr}}_{1x}\left(Y\right)\neq {\mathrm{Pr}}_{2x}\left(Y\right)$, there exists an instantiation (y) such that ${\mathrm{Pr}}_{1x}\left(y\right)\neq {\mathrm{Pr}}_{2x}\left(y\right)$. Without losing generality, assume ${\mathrm{Pr}}_{1x}\left(y\right)>{\mathrm{Pr}}_{2x}\left(y\right)$. Since ${\mathrm{Pr}}_{1x}\left(y\right)$ is computed as the marginal probability of y in the mutilated CBN for $\langle G,F_{1}\rangle$, it can be expressed in the form of a *network polynomial* as shown in [38,47]. If we treat the CPT entries of $f_{Z}\left(Z|p\right)$ as unknown, then we can write ${\mathrm{Pr}}_{1x}\left(y\right)$ as follows:
  $${\mathrm{Pr}}_{1x}\left(y\right)=\alpha_{0}+\alpha_{1}f_{Z}\left(z_{1}|p\right)+\cdots +\alpha_{k}f_{Z}\left(z_{k}|p\right)$$
  where $\alpha_{0},\alpha_{1},\cdots ,\alpha_{k}$ are constants and $z_{1},\cdots ,z_{k}$ are the states of variable *Z*. Similarly, we can write ${\mathrm{Pr}}_{2x}\left(y\right)$ as follows:
  $${\mathrm{Pr}}_{2x}\left(y\right)=\beta_{0}+\beta_{1}g_{Z}\left(z_{1}|p\right)+\cdots +\beta_{k}g_{Z}\left(z_{k}|p\right)$$

Let $\alpha_{i}$ be the *maximum* value among $\alpha_{1},\cdots ,\alpha_{k}$ and $\beta_{j}$ be the *minimum* value among $\beta_{1},\cdots ,\beta_{k}$; our construction method assigns $f_{Z}^{\prime }\left(z_{i}|p\right)=1$ and $g_{Z}^{\prime }\left(z_{j}|p\right)=1$. By construction, it is guaranteed that ${\mathrm{Pr}}_{1x}^{\prime }\left(y\right)-{\mathrm{Pr}}_{2x}^{\prime }\left(y\right)\geq {\mathrm{Pr}}_{1x}\left(y\right)-{\mathrm{Pr}}_{2x}\left(y\right)>0$, where ${\mathrm{Pr}}_{1x}^{\prime }\left(y\right)$ and ${\mathrm{Pr}}_{2x}^{\prime }\left(y\right)$ denote the causal effects under the updated CPTs $f_{Z}^{\prime }\left(Z|p\right)$ and $g_{Z}^{\prime }\left(Z|p\right)$, respectively. We repeat the above procedure for all p, where ${\mathrm{Pr}}_{1}\left(p\right)=0$, which yields the new CBNs ($\langle G,F_{1}^{\prime }\rangle$ and $\langle G,F_{2}^{\prime }\rangle$) in which $f_{Z}^{\prime }\left(Z|p\right)$ and $g_{Z}^{\prime }\left(Z|p\right)$ are functional whenever ${\mathrm{Pr}}_{1}\left(p\right)=0$. Next, we show that $F_{1}^{\prime }$ and $F_{2}^{\prime }$ (with the updated $f_{Z}^{\prime }$ and $g_{Z}^{\prime }$) constitute an example of unidentifiability. ${\mathrm{Pr}}_{1x}^{\prime }\left(Y\right)\neq {\mathrm{Pr}}_{2x}^{\prime }\left(Y\right)$, since ${\mathrm{Pr}}_{1x}^{\prime }\left(y\right)>{\mathrm{Pr}}_{2x}^{\prime }\left(y\right)$ for the particular instantiation (y). We are left to show that the distributions of ${\mathrm{Pr}}_{1}^{\prime }$ and ${\mathrm{Pr}}_{2}^{\prime }$ induced by $\langle G,F_{1}^{\prime }\rangle$ and $\langle G,F_{2}^{\prime }\rangle$ are the same over the observed variables (V). Consider each instantiation (v) of V and p of P, where p is consistent with v. If ${\mathrm{Pr}}_{1}\left(p\right)=0$, then ${\mathrm{Pr}}_{1}^{\prime }\left(p\right)={\mathrm{Pr}}_{2}^{\prime }\left(p\right)=0$ according to Lemma A3; thus, ${\mathrm{Pr}}_{1}^{\prime }\left(v\right)={\mathrm{Pr}}_{2}^{\prime }\left(v\right)=0$. Otherwise, ${\mathrm{Pr}}_{1}^{\prime }\left(v\right)={\mathrm{Pr}}_{1}\left(v\right)={\mathrm{Pr}}_{2}\left(v\right)={\mathrm{Pr}}_{2}^{\prime }\left(v\right)$, since none of the CPT entries consistent with v was modified.

**Second Step:** We construct $\langle G^{\prime \prime },F_{1}^{\prime \prime }\rangle$ and $\langle G^{\prime \prime },F_{2}^{\prime \prime }\rangle$ from $\langle G,F_{1}^{\prime }\rangle$ and $\langle G,F_{2}^{\prime }\rangle$, respectively, by introducing an auxiliary root parent for *Z* and assigning a functional CPT for *Z*. We add a root variable, denoted as *R*, to be an auxiliary parent of *W*, which specifies all possible functions from $P^{*}$ to *Z*, where $P^{*}$ contains all instantiations of $p^{*}$, where ${\mathrm{Pr}}_{1}^{\prime }\left(p^{*}\right)={\mathrm{Pr}}_{2}^{\prime }\left(p^{*}\right)>0$. Each state (*r*) of *R* corresponds to a function ($\varphi_{r}$), where $\varphi_{r}\left(p^{*}\right)$ is mapped to some state of *Z* for each instantiation ($p^{*}$). Thus, the *R* variable has ${\left|Z\right|}^{|P^{*}|}$ states, since there is total of ${\left|Z\right|}^{|P^{*}|}$ possible functions from $P^{*}$ to *Z*. For each instantiation (z,r,p), if $p\in P^{*}$, we define $f_{Z}^{\prime \prime }\left(z|r,p\right)=1$ if $z=\varphi_{r}\left(p\right)$ and $f_{Z}^{\prime \prime }\left(z|r,p\right)=0$ otherwise. If $p\notin P^{*}$, we define $f_{Z}^{\prime \prime }\left(z|r,p\right)=f_{Z}^{\prime }\left(z|p\right)$. The CPT for *R* is assigned as $f_{R}^{\prime \prime }\left(r\right)=\prod_{p\in P^{*}}f_{Z}^{\prime }\left(\varphi_{r}\left(p\right)|p\right)$. Moreover, we remove all the states (*r*) of *R*, where $f_{R}^{\prime \prime }\left(r\right)=0$, which ensures $f_{R}^{\prime \prime }\left(r\right)>0$ for all remaining *r*. We assign CPT $g_{Z}^{\prime \prime }$ in $F_{2}^{\prime \prime }$ in a similar way. Note that $f_{R}^{\prime \prime }=g_{R}^{\prime \prime }$, since $f_{Z}^{\prime }\left(\varphi_{r}\left(p\right)|p\right)={\mathrm{Pr}}_{1}^{\prime }\left(\varphi_{r}\left(p\right)|p\right)={\mathrm{Pr}}_{2}^{\prime }\left(\varphi_{r}\left(p\right)|p\right)=g_{Z}^{\prime }\left(\varphi_{r}\left(p\right)|p\right)$ for each $p\in P^{*}$ (where ${\mathrm{Pr}}_{1}^{\prime }\left(p\right)={\mathrm{Pr}}_{2}^{\prime }\left(p\right)>0$).

Next, we show that $\langle G^{\prime \prime },F_{1}^{\prime \prime }\rangle$ and $\langle G^{\prime \prime },F_{2}^{\prime \prime }\rangle$ constitute unidentifiability. One key observation is that $f_{T}^{\prime }\left(t|p\right)=\sum_{r}f_{R}^{\prime \prime }\left(r\right)f_{T}^{\prime \prime }\left(t|p,r\right)$ and $g_{T}^{\prime }\left(t|p\right)=\sum_{r}g_{R}^{\prime \prime }\left(r\right)g_{T}^{\prime \prime }\left(t|p,r\right)$. Consider each instantiation (v,r) that contains an instantiation (p) of P and the state (*z*) of *Z*. Suppose ${\mathrm{Pr}}_{1}^{\prime }\left(p\right)=0$; then, ${\mathrm{Pr}}_{1}^{\prime \prime }\left(p\right)=0$, since the marginal distribution over V is preserved in ${\mathrm{Pr}}_{1}^{\prime \prime }$. Hence, ${\mathrm{Pr}}_{1}^{\prime \prime }\left(v,r\right)={\mathrm{Pr}}_{2}^{\prime \prime }\left(v,r\right)=0$. Suppose ${\mathrm{Pr}}_{1}^{\prime }\left(p\right)\neq 0$; then, ${\mathrm{Pr}}_{1}^{\prime \prime }\left(v,r\right)=\left(\prod_{V\in V\setminus \{Z\}}f_{V}^{\prime }\right)\cdot f_{R}^{\prime \prime }\left(r\right)$ when $z=\varphi_{r}\left(p\right)$ and ${\mathrm{Pr}}_{1}^{\prime \prime }\left(v,r\right)=0$ otherwise. Similarly, ${\mathrm{Pr}}_{2}^{\prime \prime }\left(v,r\right)=\left(\prod_{V\in V\setminus \{Z\}}g_{V}^{\prime }\right)\cdot g_{R}^{\prime \prime }\left(r\right)$ when $z=\varphi_{r}\left(p\right)$ and ${\mathrm{Pr}}_{2}^{\prime \prime }\left(v,r\right)=0$ otherwise. In both cases, ${\mathrm{Pr}}_{1}^{\prime \prime }\left(v,r\right)={\mathrm{Pr}}_{2}^{\prime \prime }\left(v,r\right)$, since $F_{1}^{\prime \prime }$ and $F_{2}^{\prime \prime }$ assign the same function ($\varphi_{r}$) for each state (*r*) of *R* and $f_{V}^{\prime }=g_{V}^{\prime }$ for all V∈V∖{Z}. With respect to ${\mathrm{Pr}}_{1x}^{\prime \prime }\left(Y\right)={\mathrm{Pr}}_{1x}^{\prime }\left(Y\right)$ and ${\mathrm{Pr}}_{2x}^{\prime \prime }\left(Y\right)={\mathrm{Pr}}_{2x}^{\prime }\left(Y\right)$, note that summing out *R* from ${\mathrm{Pr}}_{1x}^{\prime \prime }\left(V,R\right)$ and ${\mathrm{Pr}}_{2x}^{\prime \prime }\left(V,R\right)$ yields ${\mathrm{Pr}}_{1x}^{\prime }\left(V\right)$ and ${\mathrm{Pr}}_{2x}^{\prime }\left(V\right)$, respectively.

**Third Step:** We construct $\langle G,F_{1}^{\prime \prime \prime }\rangle$ and $\langle G,F_{2}^{\prime \prime \prime }\rangle$ from $\langle G^{\prime \prime },F_{1}^{\prime \prime }\rangle$ and $\langle G^{\prime \prime },F_{2}^{\prime \prime }\rangle$, respectively, by merging the auxiliary root variable (*R*) with an observed parent (*T*) of *Z*. We merge *R* and *T* into a new node ($T^{\prime }$) and substitute it for *T* in *G*, i.e., $T^{\prime }$ has the same parents and children as *T* in *G*. Specifically, $T^{\prime }$ is constructed as the Cartesian product of *R* and *T*. Each state of $T^{\prime }$ can be represented as (r,t), where *r* is a state of *R* and *t* is a state of *T*. We then assign the CPT ($f_{T^{\prime }}^{\prime \prime \prime }\left(T^{\prime }|P_{T}\right)$) in $F_{1}^{\prime \prime \prime }$ as follows. For each parent instantiation ($p_{T^{\prime }}$) and each state (r,t) of $T^{\prime }$, $f_{T^{\prime }}^{\prime \prime \prime }\left(\left(r,t\right)|p_{T^{\prime }}\right)=f_{R}^{\prime \prime }\left(r\right)f_{T}^{\prime \prime }\left(t|p_{T^{\prime }}\right)$. For each child (*C*) of $T^{\prime }$ that has parents ($P_{C}$) (excluding $T^{\prime }$), the CPT for *C* in $F_{1}^{\prime \prime \prime }$ is assigned as $f_{C}^{\prime \prime \prime }\left(c|p_{C},\left(r,t\right)\right)=f_{C}^{\prime \prime }\left(c|p_{C},t\right)$. Similarly, we assign the CPTs ($g_{T^{\prime }}^{\prime \prime \prime }$ and $g_{C}^{\prime \prime \prime }$) in $F_{2}^{\prime \prime \prime }$. The distributions over observed variables are preserved, since there is a one-to-one correspondence between the instantiations over V∪{R} in $\langle G^{\prime \prime },F_{1}^{\prime \prime }\rangle$ and the instantiations over V in $\langle G,F_{1}^{\prime \prime \prime }\rangle$. Next, we consider the causal effect. Suppose *T* is neither a treatment nor an outcome variable; then, merging does not affect the causal effect in a one-to-one correspondence between instantiations and ${\mathrm{Pr}}_{1x}^{\prime \prime \prime }\left(Y\right)={\mathrm{Pr}}_{1x}^{\prime \prime }\left(Y\right)\neq {\mathrm{Pr}}_{2x}^{\prime \prime }\left(Y\right)={\mathrm{Pr}}_{2x}^{\prime \prime \prime }\left(Y\right)$. Suppose *T* is an outcome variable; since ${\mathrm{Pr}}_{1x}^{\prime \prime }\left(Y\right)\neq {\mathrm{Pr}}_{2x}^{\prime \prime }\left(Y\right)$, there exists an instantiation $\left(y^{\prime },r,t\right)$ where $Y^{\prime }=Y\setminus \left\{T\right\}$ such that ${\mathrm{Pr}}_{1x}^{\prime \prime }\left(y^{\prime },r,t\right)\neq {\mathrm{Pr}}_{2x}^{\prime \prime }\left(y^{\prime },r,t\right)$. This implies ${\mathrm{Pr}}_{1x}^{\prime \prime \prime }\left(y^{\prime },\left(r,t\right)\right)\neq {\mathrm{Pr}}_{2x}^{\prime \prime \prime }\left(y^{\prime },\left(r,t\right)\right)$ for the particular instantiation ($y^{\prime }$) and the state (r,t) of $T^{\prime }$. Suppose *T* is the treatment variable (*X*); since ${\mathrm{Pr}}_{1x}^{\prime \prime }\left(Y\right)\neq {\mathrm{Pr}}_{2x}^{\prime \prime }\left(Y\right)$, there exists an instantiation (y,r) such that ${\mathrm{Pr}}_{1x}^{\prime \prime }\left(y,r\right)\neq {\mathrm{Pr}}_{2x}^{\prime \prime }\left(y,r\right)$. Moreover, ${\mathrm{Pr}}_{1x}^{\prime \prime }\left(r\right)={\mathrm{Pr}}_{1}^{\prime \prime }\left(r\right)={\mathrm{Pr}}_{2}^{\prime \prime }\left(r\right)={\mathrm{Pr}}_{2x}^{\prime \prime }\left(r\right)>0$ (otherwise, ${\mathrm{Pr}}_{1x}^{\prime \prime }\left(y,r\right)={\mathrm{Pr}}_{2x}^{\prime \prime }\left(y,r\right)=0$). This implies ${\mathrm{Pr}}_{1x}^{\prime \prime }\left(y|r\right)\neq {\mathrm{Pr}}_{2x}^{\prime \prime }\left(y|r\right)$. Since *R* is a root in the mutilated CBN, ${\mathrm{Pr}}_{1(xr)}^{\prime \prime }\left(y\right)={\mathrm{Pr}}_{1x}^{\prime \prime }\left(y|r\right)\neq {\mathrm{Pr}}_{2x}^{\prime \prime }\left(y|r\right)={\mathrm{Pr}}_{2(xr)}^{\prime \prime }\left(y\right)$. We now consider treatment $do(T^{\prime }=\left(r,x\right))$ on *G* instead of treatment do(R=r,X=x) on $G^{\prime \prime }$. We have ${\mathrm{Pr}}_{1((r,x))}^{\prime \prime \prime }\left(y\right)={\mathrm{Pr}}_{1(r,x)}^{\prime \prime }\left(y\right)\neq {\mathrm{Pr}}_{2(r,x)}^{\prime \prime }\left(y\right)={\mathrm{Pr}}_{2((r,x))}^{\prime \prime \prime }\left(y\right)$ for the particular state (r,x) of $T^{\prime }$. Moreover, ${\mathrm{Pr}}_{1}^{\prime \prime \prime }\left(\left(r,x\right)\right)={\mathrm{Pr}}_{1}^{\prime \prime }\left(r,x\right)={\mathrm{Pr}}_{1}^{\prime \prime }\left(r\right){\mathrm{Pr}}_{1}^{\prime \prime }\left(x\right)>0$ according to the positivity assumption of ${\mathrm{Pr}}_{1}^{\prime \prime }\left(x\right)$. Thus, the positivity still holds for ${\mathrm{Pr}}_{1}^{\prime \prime \prime }$ and, similarly, for ${\mathrm{Pr}}_{2}^{\prime \prime \prime }$. □

**Proof of Theorem** **5.** Let $H=V^{\prime }\setminus V$ be a set of hidden functional variables that are functionally determined by V. According to Theorem 2, the set is F-identifiable with respect to $\langle G,V,C_{V},W\rangle$ iff it isF-identifiable with respect to $\langle G^{\prime },V,C_{V},W\setminus H\rangle$, where $G^{\prime }$ is the result of functionally eliminating H from *G*. By construction, every variable in H has parents in $V^{\prime }$. Hence, according to Theorem 3, a variable is F-identifiable with respec t to $\langle G^{\prime },V,C_{V},W\setminus H\rangle$ iff it is F-identifiable with respect to $\langle G,V^{\prime },C_{V},W\rangle$. If we consider each functional variable (W∈W), it is either in $V^{\prime }$ or has a parent that is not in $V^{\prime }$ (otherwise, *W* would have been added to $V^{\prime }$). According to Lemma A4, a functional variable is F-identifiable with respect to $\langle G,V^{\prime },C_{V},W\rangle$ iff it is identifiable with respect to $\langle G,V^{\prime },C_{V}\rangle$. □
